# ICOSL expression in human bone marrow-derived mesenchymal stem cells promotes induction of regulatory T cells

**DOI:** 10.1038/srep44486

**Published:** 2017-03-14

**Authors:** Hyun-Joo Lee, Si-Na Kim, Myung-Shin Jeon, TacGhee Yi, Sun U. Song

**Affiliations:** 1 Translational Research Center, Inha University School of Medicine, Incheon, Republic of Korea; 2 Drug Development Program, Department of Biomedical Sciences, Inha University School of Medicine, Incheon, Republic of Korea; 3 SCM Lifesciences Co. Ltd., Incheon, Republic of Korea; 4 SunCreate Co. Ltd., Yangju, Republic of Korea

## Abstract

Mesenchymal stem cells (MSCs) can modulate lymphocyte proliferation and function. One of the immunomodulatory functions of MSCs involves CD4^+^CD25^+^FoxP3^+^ regulatory T cells (Tregs), which negatively regulate inflammatory responses. MSC-mediated Treg induction is supposed to be regulated by mechanisms requiring both soluble and cell contact-dependent factors. Although the involvement of soluble factors has been revealed, the contact-dependent mechanisms in MSC-mediated Treg induction remain unclear. We attempted to identify molecule(s) other than secreted factors that are responsible for MSC-mediated Treg induction and to uncover the underlying mechanisms. Under *in vitro* Treg-inducing conditions, ICOSL expression in MSCs coincided with Treg induction in co-cultures of MSCs with CD4^+^ T cells. When cultured in a transwell plate, MSCs failed to induce Tregs. Neutralization or knockdown of ICOSL significantly reduced Tregs and their IL-10 release. ICOSL overexpression in MSCs promoted induction of functional Tregs. ICOSL-ICOS signaling promoted Treg differentiation from CD4^+^ T cells through activation of the phosphoinositide 3-kinase-Akt pathway. MSCs primed with Interleukin-1β significantly induced Tregs through ICOSL upregulation. We demonstrated that the Treg-inducing activity of MSCs is proportionate to their basal ICOSL expression. This study provides evidence that ICOSL expression in human MSCs plays an important role in contact-dependent regulation of MSC-mediated Treg induction.

Stem cells are multipotent, indicating that they can transdifferentiate to other cell types upon appropriate induction. Mesenchymal stem cells (MSCs), unlike embryonic stem cells (ESCs), induced pluripotent stem cells (iPSCs), and hematopoietic stem cells (HSCs), can reduce exacerbated inflammation due to their intrinsic immunomodulatory properties. They are known to improve pathological conditions by alleviating inflammatory immune responses in a variety of inflammatory diseases, including graft-versus-host disease (GVHD), colitis, pancreatitis, atopic dermatitis, and diabetes^1^,^2^,^3^,^4^,^5^. MSCs can modulate the functions of various immune cell types including lymphocytes, dendritic cells, and macrophages. MSCs are known to suppress activated lymphocytes in various ways^2^,^3^,^5^,^6^. MSC-driven suppression of immune responses and inflammation also involves a CD4^+^ T cell subset of regulatory T cells (Tregs)^7^. FoxP3-expressing Tregs among CD4^+^CD25^+^ T cells suppress deleterious immune responses and inflammation by actively inhibiting CD4^+^ T cells, CD8^+^ T cells, dendritic cells (DCs), natural killer cells (NKs), and B cells in a cell-cell contact and dose-dependent manner^8^. They also accumulate in tumor environments to protect developing tumor cells from immune attack, and their frequencies correlate with poor prognosis^9^. When activated by T cell receptor (TCR) stimulation, Tregs express co-stimulatory molecules such as CD28 and inducible T cell co-stimulator (ICOS) for their proliferation, survival, and activity.

ICOSL belongs to the B7 family of co-stimulatory molecules and shares sequence similarity with CD80 and CD86^10^. ICOSL does not interact with CD28 or cytotoxic T lymphocyte-associated protein 4 (CTLA-4) despite its sequence homology with them, but rather binds to its receptor ICOS. Blocking ICOS-ICOSL interaction exacerbates experimental allergic encephalomyelitis, suggesting that its signaling negatively regulates unfavorable immune responses^11^. In tumor microenvironments, Tregs protect tumors from immune cells. Tregs from cancer patients tend to show high ICOS expression and display stronger suppressive functions compared to Tregs from normal donors^12^,^13^,^14^. ICOS signaling is required for active suppression by Tregs^12^. ICOS ligand (ICOSL) expressed by antigen-presenting cells, epithelial cells, and tumor cells, is reported to directly drive Treg expansion and activation^15^. Recently, significant upregulation of ICOSL in MSCs has been observed under inflammatory conditions^16^. However, there are no reports regarding the functional role of ICOSL in MSCs.

Accumulating evidence indicates that MSCs promote Treg induction to negatively regulate T cell activation^7^,^17^,^18^. However, how MSCs affect CD4^+^ T cells to produce anergic FoxP3^+^ Tregs, remains unknown. Despite an unclear molecular mechanism of action, MSC-mediated Treg induction is likely controlled by a mechanism requiring both soluble factors and cell contact-dependent events. Selmani *et al*. demonstrated that the human leukocyte antigen-G5 (HLA-G5) secreted from MSCs contributes to the expansion of FoxP3-expressing Tregs^19^. Afterwards, English *et al*. suggested the role of soluble factors secreted from MSCs in Treg induction. They showed that prostaglandin E2 (PGE2) and transforming growth factor-β1 (TGF-β1) play important roles in MSC-mediated induction of CD4^+^CD25^+^FoxP3^+^ Tregs^20^. They also demonstrated a reliable result for the involvement of a cell contact-dependent mechanism in Treg induction. However, they did not elucidate the molecular identity of the direct contact between MSCs and T cells for Treg induction, suggesting that other unknown molecular regulator(s) might be associated with the contact-dependent mechanism underlying this event.

Based on these findings, we hypothesized that ICOSL expressed in MSCs may play a critical role in MSC-mediated Treg induction, by signaling via ICOS to promote Treg differentiation. In this study, we investigated whether ICOSL may be functionally associated with MSC-mediated immunosuppression. We examined the functional role of ICOSL in MSC-mediated induction of CD4^+^CD25^+^FoxP3^+^ Tregs and revealed the underlying molecular mechanism. We provided evidence that inflammation-induced ICOSL expression in MSCs promotes Treg induction. Furthermore, we investigated the priming effect of interleukin-1β (IL-1β) on ICOSL upregulation to enhance the functional activity of MSCs for Treg induction.

## Results

### ICOSL expression in MSCs coincides with the induction of functional Tregs

**Figure 1:**
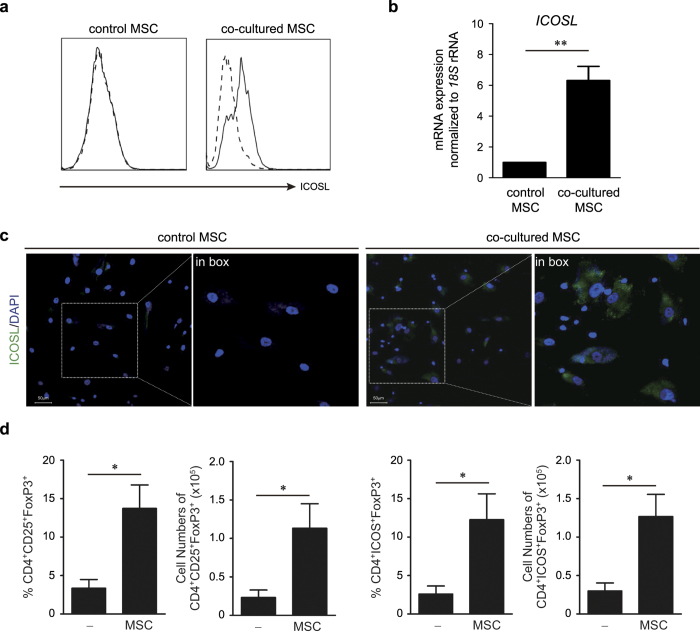
ICOSL expression in MSCs and MSC-mediated induction of Tregs. (**a**) Flow cytometric analysis revealed that ICOSL was induced in BM-derived human MSCs upon co-cultured with human CD4^+^ T cells purified from PBMCs under *in vitro* Treg induction conditions. Dashed line: isotype-matched control antibody, solid line: anti-ICOSL antibody. (**b**) Relative quantitation of *ICOSL* transcripts by qPCR showing *ICOSL* upregulation in co-cultured MSCs. *ICOSL* expression in resting MSCs (control) was insignificant. Data represent the mean ± standard deviation from three independent experiments. **P = 0.001. (**c**) Immunofluorescence staining for ICOSL expression between control and co-cultured MSCs. DAPI was used for nuclear staining. Scale bars, 50 μm. (**d**) Expression of FoxP3, CD25, and ICOS in CD4^+^ T cells was determined by flow cytometry. Under *in vitro* Treg induction, more CD25^+^FoxP3^+^ Treg cells were generated from CD4^+^ T cells in co-culture with MSCs. ICOS expression was concurrently increased in these cells. Each result is presented in two different ways, percentages and cell numbers. Data are the average of three independent experiments. Statistical significance was *P < 0.05.

Previously, we reported drastic ICOSL induction in human MSCs stimulated with inflammatory cytokines but its functional role with respect to stem cell properties was not investigated^16^. Recently, ICOSL-expressing melanoma cells were reported to stimulate Treg induction, prompting us to question whether ICOSL in MSCs promotes Treg induction or conversion^15^. MSCs isolated from human bone marrow (BM) were used in this study. They were positive for typical MSC markers, including CD29, CD44, CD73, CD90, CD105, human leu cocyte antigen (HLA) class I, and the pluripotency marker Oct4, but were negative for CD34, CD45, and HLA-DR (Supplementary Fig. S1a). They did not express co-stimulatory molecules like CD80 and CD86 (Supplementary Fig. S1a). They suppressed phytohemagglutinin (PHA)-stimulated peripheral blood mononuclear cell (PBMC) proliferation in a cell number-dependent manner (Supplementary Fig. S1b). First, we simply tested our hypothesis by comparing the effects of these MSCs on FoxP3-expressing Tregs. Tregs were induced *in vitro* by stimulating CD4^+^ T cells purified from human PBMCs with anti-CD3, anti-CD28, interleukin-2 (IL-2), TGF-β1, and all-trans-retinoic acid to generate Tregs (Supplementary Fig. S1c). After co-culturing CD4^+^ T cells with or without MSCs for 24–72 h under these conditions, Treg phenotypes were analyzed. Consistent with previous studies, co-culture with MSCs significantly increased CD25^+^FoxP3^+^ Treg induction from CD4^+^ cells (Supplementary Fig. S1d,e). During co-culture, ICOSL was significantly upregulated in MSCs at both mRNA and protein levels (Fig. 1a–c). Since ICOSL binds to its receptor, ICOS on activated lymphocytes^21^,^22^, we analyzed ICOS expression in Tregs. MSC-induced Tregs showed higher ICOS expression (Fig. 1d, Supplementary Fig. S2a). They were further characterized by downregulation of CD127 (Supplementary Fig. S1e). We next examined whether MSC-induced Tregs are functional. Co-culture of induced Tregs with carboxyfluorescein succinimidyl ester (CFSE)-labeled PBMCs, inhibited PBMC proliferation, indicating the suppressive function of MSC-induced Tregs (Supplementary Fig. S2b). Additionally, these Tregs themselves were anergic to TCR stimulation (Supplementary Fig. S2c).

### MSCs-mediated Treg induction depends on cell-cell contact

**Figure 2:**
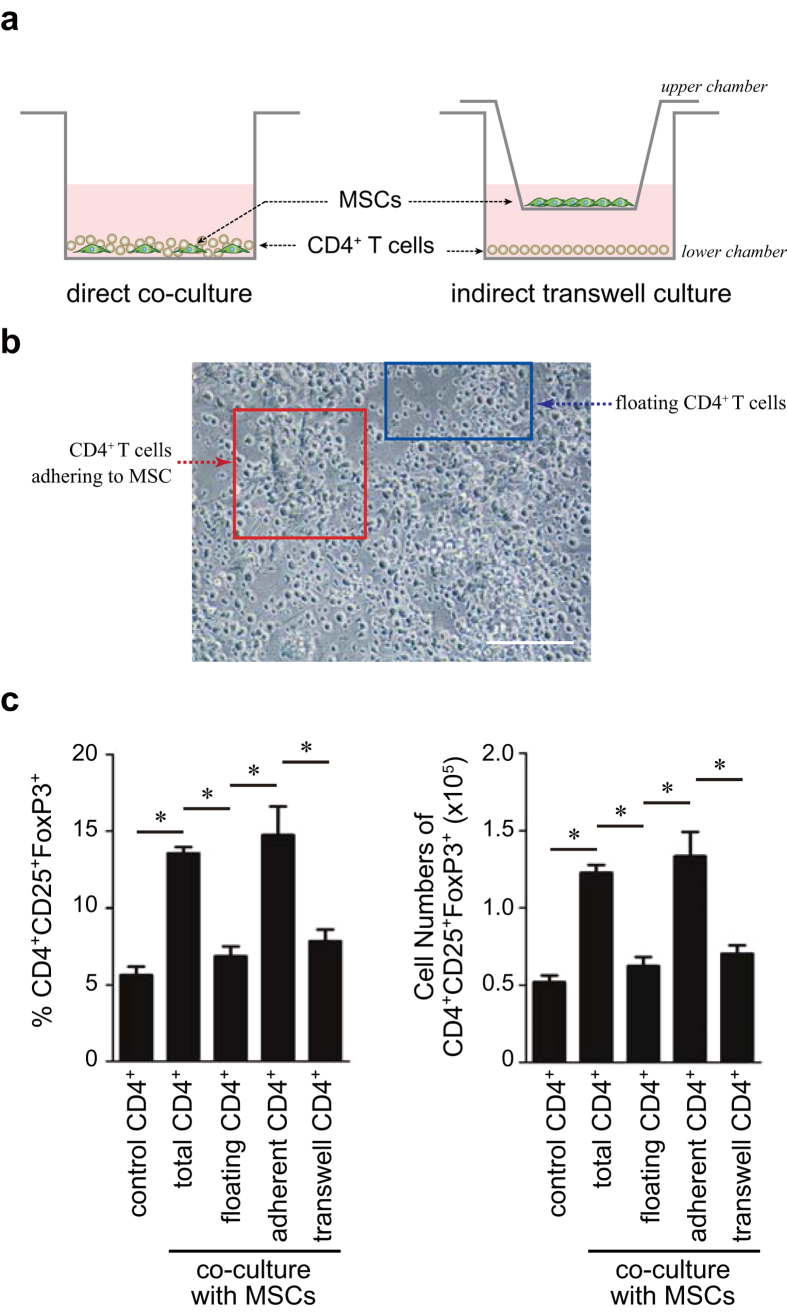
Comparison of Treg induction between direct co-culture and indirect transwell culture conditions. (**a**) A schematic of the experiments. In transwell cultures, MSCs (1 × 10^5^) were seeded on the upper chamber and CD4^+^ T cells (1 × 10^6^) were in the lower chamber. (**b**) An image of the direct co-culture of MSCs with CD4^+^ T cells under a light microscope. Blue box indicates non-adherent floating T cells while red box shows T cells adhering MSCs. Scale bar, 200 μm. (**c**) Total CD4^+^ T cells, only floating T cells, MSC-adherent T cells, or T cells cultured in a transwell plate were subjected to flow cytometric analysis for the CD25^+^FoxP3^+^ Treg phenotype. Non-adherent floating and adherent CD4^+^ T cells were separately harvested from the same co-cultures. Each result is presented in two different ways, percentages and cell numbers. Data are the average of three independent experiments. Statistical significance was *P < 0.05.

Cell-cell contact between MSCs and T cells appeared to be essential for MSC-mediated Treg induction^20^. We examined whether floating CD4^+^ T cells that were not in firm contact with the plastic-adherent MSCs in direct co-culture, exhibited Treg phenotypes (Fig. 2a). We simply collected non-adherent floating CD4^+^ T cells from direct co-cultures by gently transferring the supernatant. Interestingly, MSC-induced CD25^+^FoxP3^+^ Tregs were not observed among these floating CD4^+^ T cells obtained from direct co-cultures (Fig. 2b,c, Supplementary Fig. S3a). In contrast to floating T cells, adherent CD4^+^ T cells in strong contact with MSCs exhibited higher Treg phenotypes (Fig. 2b,c, Supplementary Fig. S3a). A transwell plate experiment was conducted to confirm the effect of cell contact on Treg induction. When MSCs and CD4^+^ T cells were separately cultured in a transwell plate (Fig. 2a), no induction of CD25^+^FoxP3^+^ Tregs was found as observed in the floating CD4^+^ T cells from the co-cultures (Fig. 2c, Supplementary Fig. S3a). These results suggested that ICOSL might play an important role in the contact-dependent mechanism for MSC-mediated Treg induction.

### ICOSL neutralization and knockdown reduces MSC-mediated Treg induction

**Figure 3:**
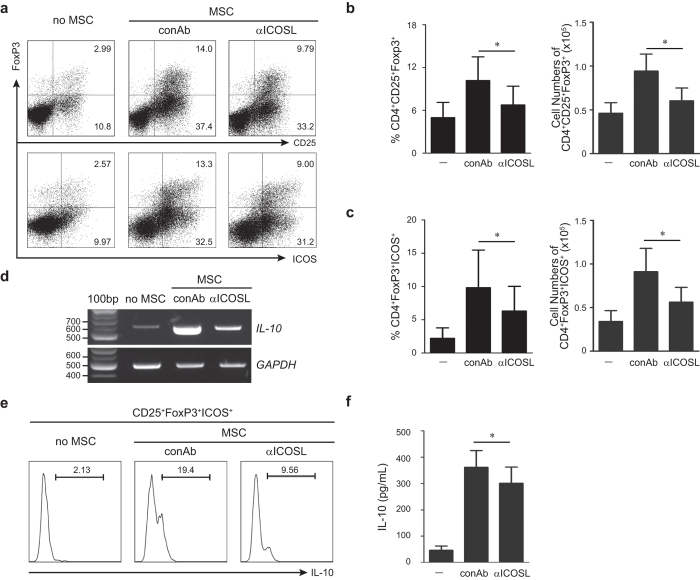
The effect of ICOSL neutralization on Treg induction. (**a**) Neutralizing anti-ICOSL antibody was added at a concentration of 10 μg/mL to the co-cultures of MSCs and CD4^+^ T cells. Flow cytometric analysis was conducted to analyze the FoxP3^+^CD25^+^ Treg population and their expression of ICOS. Dot blots are representative of three independent experiments. (**b**,**c**) The effect of ICOSL neutralization on FoxP3^+^CD4^+^CD25^+^ Treg induction was shown (**b**). Their expression of ICOS also was analyzed (**c**). Data are the mean ± standard deviation from three or more independent experiments. Each result is presented in two different ways, percentages and cell numbers. *P < 0.05. (**d**) Semi-quantitative RT-PCR showing the effect of ICOSL neutralization on IL-10 expression in MSCs. 100 bp, 100 base pair DNA ladder. (**e**) IL-10 production by CD4^+^ T cells was determined by flow cytometry upon addition of anti-ICOSL antibody to the co-cultures. (**f**) Under the condition of ICOSL neutralization, the amount of secreted IL-10 in the conditioned medium was determined by ELISA. *P = 0.03.

**Figure 4:**
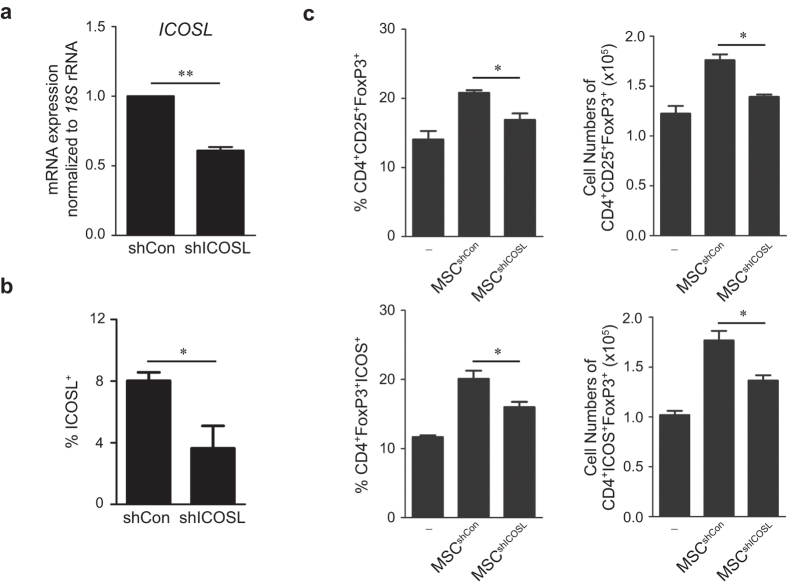
Effect of ICOSL knockdown in MSCs on Treg induction. (**a**) MSCs were infected with lentivirus expressing control shRNA (shCon) or ICOSL-specific shRNA (shICOSL). ICOSL knockdown in MSCs was assessed by qPCR. Data are the mean ± standard deviation from three independent experiments. **P = 0.008. (**b**) ICOSL-expressing MSCs were determined by flow cytometry. Data are the mean ± standard deviation from three independent experiments. *P = 0.013. (**c**) When CD4^+^ T cells were co-cultured with ICOSL knockdown MSCs (MSCsh^ICOSL^), FoxP3^+^Cd25^+^ Tregs were significantly decreased compared co-cultures with control MSCs (MSC^shCon^). Each result is presented in two different ways, percentages and cell numbers. Data are the average of three independent experiments. Statistical significance was *P < 0.05.

To evaluate the pivotal role of ICOSL as a putative Treg inducer, ICOSL was neutralized in MSCs and CD4^+^ T cell co-cultures. Treatment with a neutralizing anti-ICOSL antibody significantly decreased the CD25^+^FoxP3^+^ Treg population (Fig. 3a,b). ICOS was reduced in these Tregs (Fig. 3a,c). Since ICOS expressing Tregs potently inhibit autoreactive T cells through suppression of antigen-presenting cells by interleukin-10 (IL-10)^23^, we examined whether ICOSL neutralization could reduce IL-10 production by Tregs. Expectedly, MSC-induced Tregs highly expressed IL-10 whereas ICOSL neutralization decreased IL-10 expression and release from Tregs (Fig. 3d–f). We then performed gene knockdown using short-hairpin RNA (shRNA)-expressing lentiviruses. MSCs infected with ICOSL-specific shRNA-expressing lentivirus (shICOSL) showed significant reduction in *ICOSL* expression (Fig. 4a). ICOSL-expressing MSCs were also substantially decreased (Fig. 4b). Consistent with the neutralization results, co-culture with ICOSL-knockdown MSCs significantly decreased induction of CD25^+^FoxP3^+^ Tregs from CD4^+^ T cells (Fig. 4c, Supplementary Fig. S3b). ICOS expression was also decreased in these Tregs (Fig. 4c, Supplementary Fig. S3b).

### ICOSL overexpression in MSCs increases Tregs

**Figure 5:**
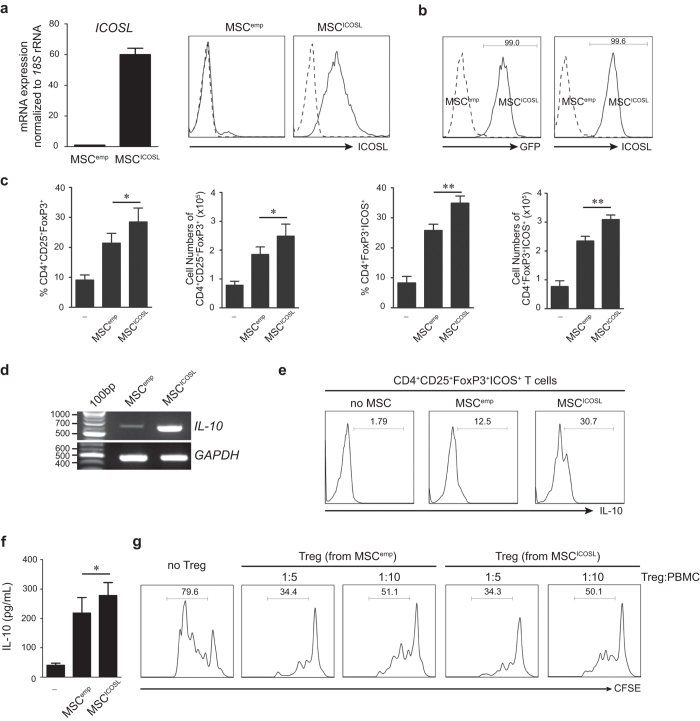
The effect of ICOSL overexpression in MSCs on Treg induction. (**a**) To confirm the Treg inducing function of ICOSL, MSCs were lentivirally transduced to constitutively express full-length human ICOSL (MSC^ICOSL^). As a control, MSCs were transduced with the empty vector (MSC^emp^). ICOSL overexpression was assessed at mRNA (left) and cell surface protein level (right). Dotted line: isotype-matched antibody, solid line: anti-ICOSL antibody. (**b**) The ICOSL-expressing vector also contains green fluorescent protein (GFP)-encoding sequences. After transduction, 99% of MSC^ICOSL^ exhibited GFP-positive signals, indicating efficient ICOSL expression. Flow cytometry using anti-ICOSL antibody confirmed ICOSL expressed on the surface of transduced MSCs. (**c**) CD4^+^ T cells were co-cultured with MSC^ICOSL^ or MSC^emp^. MSC^ICOSL^-induced CD4^+^CD25^+^FoxP3^+^ Tregs were significantly increased. ICOS-expressing Tregs were also increased upon co-culture with MSC^ICOSL^. Each result is presented in two different ways, percentages and cell numbers. Data are the mean ± standard deviation from three independent experiments. Statistical significance was *P < 0.05. (**d**) The effect of MSC^ICOSL^ on IL-10 expression in Tregs. Co-culture with MSC^ICOSL^ markedly increased IL-10 expression in the co-cultured CD4^+^ T cells. (**e**) Flow cytometric analysis was performed to examine IL-10 expression in CD25^+^Foxp3^+^ICOS^+^ population of CD4^+^ T cells after co-culture with MSC^emp^ or MSC^ICOSL^. Co-culture with MSC^ICOSL^ increased the intracellular IL-10 producing CD25^+^Foxp3^+^ICOS^+^ Tregs from CD4^+^ T cells 2.5-fold than those co-cultured with MSC^emp^. (**f**) Quantitation of IL-10 level by ELISA revealed a significant increase in IL-10 secretion when CD4^+^ T cells were co-cultured with MSC^ICOSL^. Data are the mean ± standard deviation from three independent experiments. *P = 0.034. (**g**) To examine whether the Tregs generated from MSC^ICOSL^ are functionally distinct, each Treg population was isolated from co-cultures with MSC^ICOSL^ or MSC^emp^. Then, equal numbers of each population were co-cultured with CFSE-labeled PBMCs followed by TCR stimulation (i.e. treatment with anti-CD3 and anti-CD28 antibodies) for 3 days. The suppressive activity of each Treg population was compared by inhibition of activated lymphocyte proliferation. The cell number ratio (Treg:PBMC) in co-cultures is indicated. The data are representative of three independent experiments.

To further verify the role of ICOSL in MSC-mediated Treg induction, we overexpressed ICOSL in MSCs by transducing them with lentiviruses carrying the full-length human *ICOSL* gene (MSC^ICOSL^). They effectively expressed ICOSL at both mRNA and cell surface protein levels in the absence of any stimulators (Fig. 5Aa,Bb). Under *in vitro* Treg induction, MSC^ICOSL^ induced more CD25^+^FoxP3^+^ Tregs from CD4^+^ T cells than those with control MSCs transduced with empty virions (MSC^emp^) (Fig. 5c, Supplementary Fig. S3c). MSC^ICSOL^-induced Tregs also expressed higher ICOS than MSC^emp^-induced Tregs (Fig. 5c, Supplementary Fig. S3c). Moreover, MSC^ICOSL^-induced Tregs produced more IL-10 compared to MSC^emp^-induced cells (Fig. 5d–f). In order to further examine whether Tregs induced by MSC^ICOSL^ are functionally distinct, each Treg population was isolated from co-cultures with MSC^ICOSL^ and MSC^emp^. Equal numbers of each Treg population was subject to co-culture with CFSE-labeled PBMCs followed by TCR stimulation. The suppressive activity of each Treg population was determined by inhibition of proliferation of activated PBMCs. Our data showed that there was no functional difference between the MSC^ICOSL^-induced and MSC^emp^-induced Tregs. Tregs isolated from co-cultures with MSC^ICOSL^ or MSC^emp^ were equally potent at suppressing CFSE-labeled PBMCs in a cell number-dependent manner (Fig. 5g). Nonetheless, since MSC^ICOSL^ produced more Tregs than normal MSCs, MSC^ICOSL^-induced Tregs collectively displayed more suppressive activity (Supplementary Fig. S3d).

### ICOSL signaling activates the PI3K-Akt pathway to promote Treg differentiation from CD4^+^ T cells

**Figure 6:**
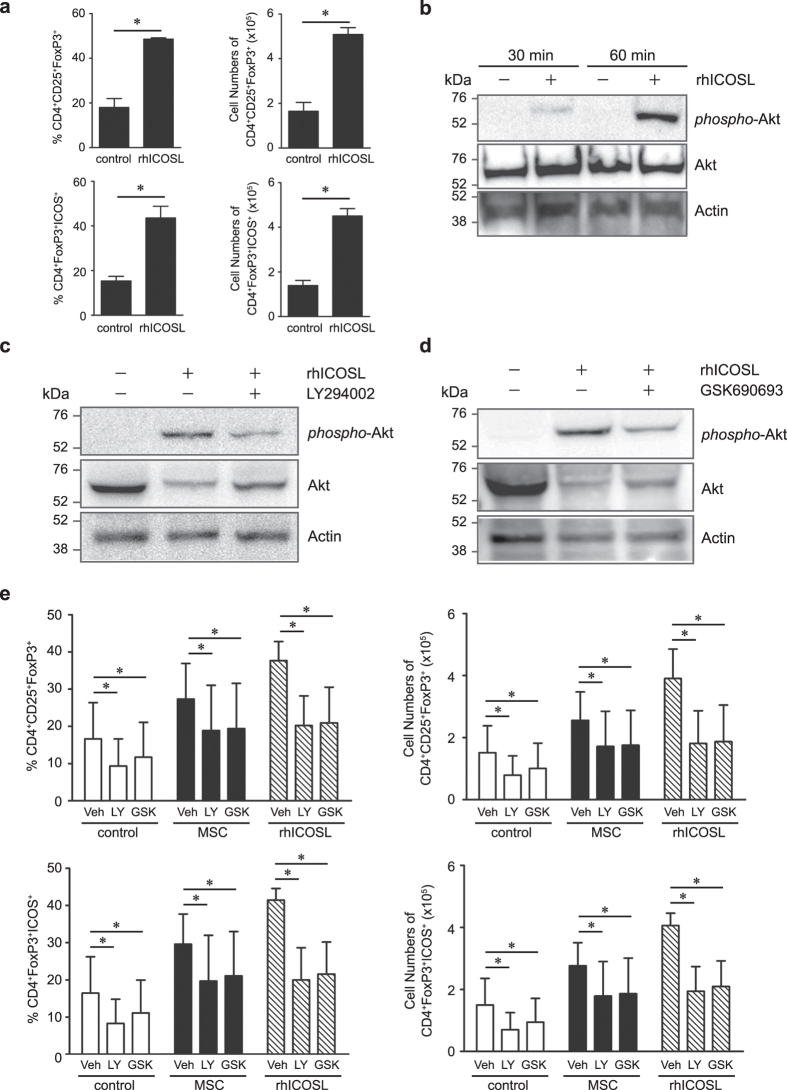
ICOSL activates the PI3K-Akt pathway to promote Treg differentiation. (**a**) Instead of MSCs, rhICOSL was coated on the plates at a concentration of 5 μg/mL. When CD4^+^ T cells were seeded and incubated under Treg inducing conditions, increased Tregs were observed with rhICOSL. Each flow cytometry data is presented in two different ways, percentages and cell numbers. Data are the average of three independent experiments. Statistical significance was *P < 0.05. (**b**) In this reaction, Akt was significantly phosphorylated within 60 min in response to rhICOSL stimulation as revealed by western blot analysis. (**c**) CD4^+^ T cells were pretreated with 5 μM LY294002 for 30 min and then seeded on rhICOSL-coated plates, incubated for 1 h under Treg inducing conditions, and then harvested for western blot analysis. Treatment with LY294002 significantly inhibited rhICOSL-induced Akt phosphorylation. (**d**) CD4^+^ T cells were pretreated with 1 μM GSK690693 for 30 min and then seeded on rhICOSL-coated plates, incubated for 1 h under Treg inducing conditions and then harvested for western blot analysis. Treatment with GSK690693 significantly inhibited rhICOSL-induced Akt phosphorylation. As controls, total Akt and actin levels were also determined. (**e**) The effects of PI3K-Akt inhibition on rhICOSL-induced Tregs were examined by incubating rhICOSL-activated CD4^+^ T cells with LY294002 (LY) or GSK690693 (GSK). After 2 days, CD4^+^ T cells were analyzed for their expression of CD25, FoxP3, or ICOS by flow cytometric analysis. Each data is presented in two different ways, percentages and cell numbers. Data are the average of three independent experiments. Statistical significance was *P < 0.05.

To define ICOSL function, the molecular signaling regulated by ICOSL-ICOS interaction in Tregs was investigated. For cell signaling analysis, we chose a simple cell system rather than a mixed co-culture system with complex heterotypic cell interactions. Instead of MSCs, recombinant human ICOSL (rhICOSL) was coated on plates at 5 μg/mL to mimic the membrane-bound ICOSL on MSCs. In the absence of MSCs, rhICOSL sufficiently induced Tregs from CD4^+^ T cells under *in vitro* Treg induction (Fig. 6a, Supplementary Fig. S4a). During this reaction, Akt was immediately phosphorylated by rhICOSL (Fig. 6b), suggesting that ICOSL activates the phosphoinositide 3-kinase (PI3K)-Akt pathway during Treg differentiation. To demonstrate the involvement of PI3K-Akt signaling in ICOSL-induced Treg differentiation, the effects of PI3K or Akt inhibition were examined. Treatment with LY294002, a PI3K inhibitor, or GSK690693, an Akt inhibitor, resulted in marked decrease in Akt phosphorylation (Fig. 6c,d). Both PI3K inhibition and Akt inhibition significantly decreased induction of Tregs by rhICOSL and MSCs (Fig. 6e, Supplementary Fig. S4b). Additionally, PI3K-Akt inhibition decreased Tregs at basal conditions (Fig. 6e, Supplementary Fig. S4b). These results suggest that the signals transmitted by ICOSL-ICOS interaction regulate Treg differentiation via the PI3K-Akt pathway.

### IL-1β priming enhances MSCs to induce Tregs by ICOSL upregulation

**Figure 7:**
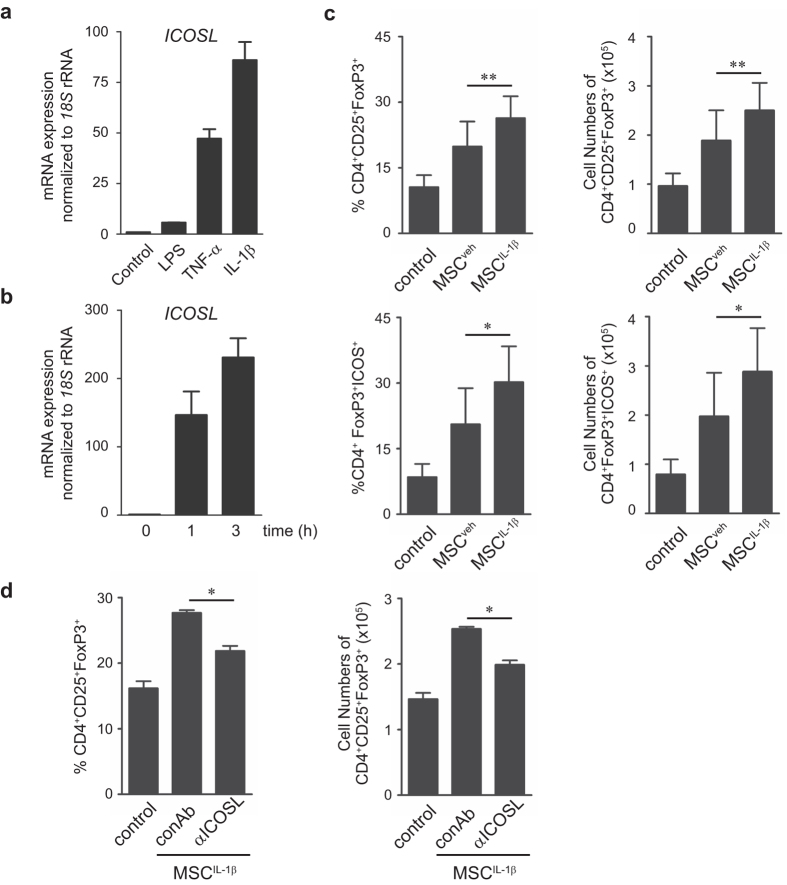
IL-1β priming enhances MSC-mediated Treg induction through ICOSL upregulation. (**a**) Relative quantitation of ICOSL expression was conducted by qPCR after MSCs were treated with 2 mg/mL LPS, 10 ng/mL TNF-α, or 10 ng/mL IL-1β for 24 h. (**b**) MSCs were treated with 10 ng/mL IL-1β for up to 3 h. qPCR was performed to determine *ICOSL* mRNA expression. (**c**) MSCs were primed with 10 ng/mL IL-1β (MSC^IL-1β^) or vehicle (MSC^veh^) for 24 h. The primed MSCs were subjected to co-culture with CD4^+^ T cells under *in vitro* Treg induction conditions. Flow cytometric analysis was performed to examine the induced Treg phenotype from CD4^+^ T cells. Each result is presented in two different ways, percentages and cell numbers. Data are the average of three independent experiments. **P < 0.01, *P < 0.05. (**d**) During Treg differentiation, CD4^+^ T cells were co-cultured with MSC^IL-1β^ in the presence of 5 μg/mL neutralizing anti-ICOSL antibody. As revealed by flow cytometry, ICOSL neutralization decreased the MSC^IL-1β^-induced Tregs. Data are the average of three independent experiments. Statistical significance was *P < 0.05.

To determine the inflammatory stimuli leading to ICOSL induction in MSCs, we tested molecules like lipopolysaccharide (LPS), tumor necrosis factor-α (TNF-α), and IL-1β. Among these, IL-1β was the most potent activator of *ICOSL* in MSCs (Fig. 7a). *ICOSL* mRNA expression was remarkably induced immediately in response to IL-1β stimulation (Fig. 7b). We hypothesized that IL-1β may enhance the Treg-inducing activity of MSCs through ICOSL upregulation. We treated MSCs with IL-1β for 24 h (MSC^IL-1β^), then harvested and co-cultured these primed cells with CD4^+^ T cells under Treg induction. MSC^IL-1β^ increased CD25^+^FoxP3^+^ Tregs to a greater extent compared to control MSCs (Fig. 7c, Supplementary Fig. S5a). Similarly, MSC^IL-1β^ increased ICOS expression in Tregs (Fig. 7c, Supplementary Fig. S5a). Neutralization with an anti-ICOSL antibody significantly decreased MSC^IL-1β^-induced Tregs, suggesting that IL-1β priming involves ICOSL signaling (Fig. 7d, Supplementary Fig. S5b). In addition, functional blockage by a neutralizing anti-IL-1β antibody reduced ICOSL expression in MSC^IL-1β^ (Supplementary Fig. S5c), suggesting that IL-1β might act as one of the ICOSL inducing factors in MSCs.

### Functional variation of MSCs in Treg induction is correlated with their basal expression of ICOSL

**Figure 8:**
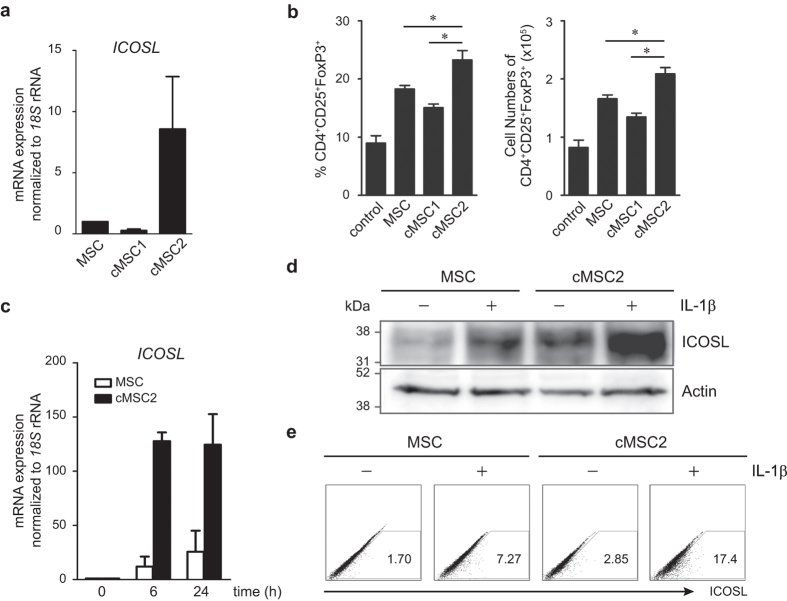
Treg-inducing activity of MSCs varies according to their basal expression of ICOSL. (**a**) Besides the nonclonal MSCs used in the above experiments, clonally selected MSCs named cMSC1 and cMSC2 were isolated from the same bone marrow sample. The basal ICOSL mRNA level among MSC, cMSC1, and cMSC2 was compared by qPCR. (**b**) Treg induction by each MSC population was compared after co-culture with CD4^+^ T cells under Treg inducing conditions. Flow cytometry data are the average of three independent experiments and presented in percentages and cell numbers. Statistical significance was *P < 0.05. (**c**) After MSC and cMSC2 were primed with 10 ng/mL IL-1β for up to 24 h, the time course expression of ICOSL mRNA was determined by qPCR. (**d**) After MSC and cMSC2 were primed with 10 ng/mL IL-1β for 24 h, ICOSL protein level was determined by western blot analysis. (**e**) After MSC and cMSC2 were primed with 10 ng/mL IL-1β for 24 h, the number ICOSL-expressing cells was analyzed by flow cytometric analysis.

Emerging evidence shows that not all MSCs are identical and MSC clones with different properties exist^24^,^25^,^26^. To examine whether MSC clones with differential basal expression of ICOSL might display differences in their functional potency to induce Tregs, we established several MSC clones by subfractionation culturing methods. We then chose two clones, cMSC1 and cMSC2 that showed differential basal expression of ICOSL. All MSCs tested in this study, including MSCs (nonclonal MSCs), cMSC1, and cMSC2, were derived from a single donor. cMSC1 expressed the lowest level and cMSC2 expressed the highest level of ICOSL mRNA (Fig. 8a). The Treg-inducing potential of each population was evaluated by co-culturing with CD4^+^ T cells under *in vitro* Treg induction. Interestingly, cMSC2 was the most potent at inducing CD25^+^FoxP3^+^Tregs (Fig. 8b, Supplementary Fig. S5d). However, cMSC1 was the least effective at Treg induction (Fig. 8b, Supplementary Fig. S5d). To examine whether ICOSL upregulation by IL-1β priming is proportionate with its basal expression, we compared IL-1β-mediated ICOSL expression between nonclonal MSCs and cMSC2. In response to IL-1β, cMSC2 expressed more ICOSL at both mRNA and protein levels compared to that in MSCs (Fig. 8c–e). We further compared ICOSL expression between adipose tissue-derived MSCs (ASCs) and MSCs derived from BM. ASCs expressed relatively low levels of ICOSL and showed low potential for Treg induction compared to BM-derived MSCs (Supplementary Fig. S5e,f). These results indicate that functional variation of MSCs in Treg induction depends on ICOSL expression.

## Discussion

In recent years, more attention has been focused on MSC immunomodulatory properties rather than their differentiation potential. MSCs exhibit immunosuppressive activities on a variety of immune cell types, including T cells, B cells, dendritic cells, macrophages, and natural killer cells^2^,^5^. This property of undifferentiated MSCs has driven the exploration of the therapeutic potential of MSCs for treating a variety of autoimmune and inflammatory diseases in clinical and translational studies^3^,^27^. However, many questions regarding the mode of action involved in these therapeutic effects remain unanswered. An exact understanding of the mechanisms by which MSCs regulate immune responses and allogeneic reactivity is essential for successful development of stem cell therapeutics. Although a number of studies have reported the immunomodulatory functions of MSCs, the underlying molecular mechanisms are only partially understood. Currently, it is considered that both soluble factors and contact-dependent mechanisms cooperate in MSC-mediated immunomodulation. Several soluble factors including PGE2, indoleamine 2,3-dioxigenase, nitric oxide, HLA-G5, TGF-β1, heme oxygenase-1, hepatocyte growth factor, and TNF-α–stimulated gene/protein 6 play important roles in creating an immunosuppressive environment^2^,^4^,^6^,^28^. Meanwhile, little is known about the contact-dependent mechanisms involved.

One of the immunomodulatory properties of MSCs is to promote Treg generation for suppressing excessive immune responses but the underlying mechanisms remain unclear. Our study addressed how MSCs promote Treg induction under inflammatory environments and aimed to specify the molecular identity and mechanisms involved. Tregs, a specialized suppressor T cell subpopulation, play a critical role in self-tolerance and immune homeostasis^29^,^30^. Both *in vitro* and *in vivo* studies show that Tregs negatively regulate effector T cell proliferation and inflammatory cytokine production^9^. Tregs are developed in the thymus and are also induced peripherally from naïve T cells. As reported earlier^31^, human MSCs significantly increased FoxP3 expression in purified human CD4^+^ T cells upon co-culture (Fig. 1d, Supplementary Fig. S2a). Consistent with previous studies^19^,^20^, direct cell-cell contact between MSCs and CD4^+^ T cells was required for the expression of Treg phenotypes (Fig. 2). Studies reporting MSC-derived soluble factors such as HLA-G4, TGF-β1, and PGE2 as Treg inducers, have also recognized that these direct cell contacts between MSCs and T cells precede the induction of these soluble factors^19^,^20^. Thus, the mechanisms of cell-contact associated with certain important molecular events that lead to Treg induction remain unknown. In this study, we demonstrated that ICOSL, a member of the B7/CD28 family of co-stimulatory molecules, expressed on MSC surface, promotes MSC-mediated Treg induction. MSCs showed highly upregulated ICOSL at both mRNA and protein levels under *in vitro* Treg inducing conditions (Fig. 1a–c). When MSCs and CD4^+^ T cells were separately cultured in a transwell plate, CD25^+^FoxP3^+^ Tregs were not induced from the CD4^+^ T cell population (Fig. 2a,c, Supplementary Fig. S3a). Moreover, a considerable number of CD25^+^FoxP3^+^ Tregs were generated from the CD4^+^ T cell fraction adhering to MSCs whereas fewer Tregs were found in the non-adherent floating cell fraction (Fig. 2b,c, Supplementary Fig. S3a). Notably, adherent CD4^+^ T cells displayed upregulated expression of ICOS at the cell periphery (Supplementary Fig. S6). Thus, transwell experiments suggested that MSC-mediated Treg induction requires cell-cell contact between MSCs and CD4^+^ T cells. Considering the functional requirement of ICOSL in Treg induction as revealed by ICOSL neutralization (Fig. 3) and knockdown (Fig. 4), ICOSL-ICOS interactions possibly contribute to this contact-dependent mechanism.

ICOSL expression in MSCs has been reported by us and other groups^16^,^32^,^33^. Here, we provide evidence that ICOSL expression in MSCs promotes CD4^+^CD25^+^FoxP3^+^ Treg induction. To our knowledge, this is the first report describing the functional role of ICOSL expressed in MSCs in the context of MSC-mediated immunomodulation. ICOSL expression in resting MSCs was insignificant but was highly induced under inflammatory conditions elicited by *in vitro* Treg cell differentiation (Fig. 1a–c). ICOSL expressed on the MSC surface appears to activate signaling by interacting with its receptor, ICOS, expressed on CD4^+^ T cells during Treg differentiation. The ICOS/ICOSL pathway has critical roles in stimulating lymphocytic responses^21^,^22^,^34^,^35^. Accumulating evidence reveals that ICOS is an important receptor for effector T cell function. ICOS is expressed on activated T cells, memory T cells, and Tregs. It is also weakly expressed in resting naïve T cells and is rapidly induced following TCR engagement and ICOSL co-stimulation on antigen presenting cells to regulate T cell activity. At early stages it can promote type 2 helper T cell (Th2) differentiation whereas in differentiated CD4^+^ T cells it can stimulate effector T cell responses, such as interferon-γ (IFN-γ) and IL-4 secretion from type 1 helper (Th1) T cells and Th2 cells, respectively^36^. ICOS is also known to regulate follicular helper T cell generation and IL-21 production^37^. In humans, ICOS mutations are correlated with common variable immunodeficiency. ICOS deficiency in humans leads to defective IL-10 and IL-17 production, impaired germinal center-affinity maturation, and isotype class switching, resulting in profound hypogammaglobuinemia^38^. Additionally, ICOS is involved in maintenance of Treg homeostasis and peripheral T cell tolerance. Its expression defines a highly suppressive effector Treg subset that produces high amounts of IL-10^23^. We observed that MSCs increased ICOS expression from FoxP3^+^ Tregs (Figs 1d, 3c and 5c). CD4^+^ T cells treated with rhICOSL resulted in increased ICOS expressing Tregs during Treg differentiation (Fig. 6a, Supplementary Fig. S4a), suggesting that ICOSL-ICOS interaction can directly promote Treg induction wherein the PI3K-Akt pathway was activated (Fig. 6b–e, Supplementary Fig. S4b). Reduced Treg differentiation by PI3K-Akt inhibitors indicated that ICOSL-ICOS interactions activate the PI3K-Akt pathway to promote Treg induction (Fig. 6). ICOS ligation provides a strong activation signal for Akt and causes sustained activation of the PI3K-Akt pathway^39^. Subsequent Akt-mediated activation of glycogen synthase kinase-3 promotes IL-10 production via Toll-like receptor ligation^40^. Thus, IL-10 production by ICOS^+^ Tregs can be regulated by the PI3K-Akt pathway during Treg differentiation. However, the functional role of this signaling pathway is controversial. Some studies have suggested that inactivation of PI3K signaling is important for Treg development^41^,^42^. Transgenic mice expressing constitutively active Akt exhibited elevated T cell activation and cytokine secretion along with symptoms of autoimmune disease^43^. In contrast, other studies have shown that PI3K signaling is required for Treg induction and function. Phosphoinositide-dependent protein kinase 1-deficient T cells exhibit reduced conversion to inducible Tregs both *in vitro* and *in vivo*^44^. Treg induction by TGF-β is reduced by pan-PI3K inhibitors^45^. Upon stable Akt activation, more inducible Tregs develop^46^. Consistent with these findings, our results support that the PI3K-Akt pathway activated by ICOSL-ICOS interactions promotes Treg induction.

Another implication of our study herein is that MSCs likely promotes Treg conversion or differentiation rather than proliferation or expansion. When we performed MSC-mediated Treg induction experiments with conventional CD4^+^ T cells after depletion of CD25^+^ cells, CD25-depleted CD4^+^ T cells produced as many Tregs as nondepleted whole CD4^+^ T cells did (Supplementary Fig. S7a–c). Another indirect evidence is as follows; we simply purified CD4^+^ T cells from 2 different PBMCs (named PBMC1 and PBMC2) without Treg depletion. At the start of the experiment, CD4^+^ T cells expressing CD25 and FoxP3 were 1.62% in PBMC1 and 3.48% in PBMC2 by flow cytometric analysis. Each CD4^+^ T cell population was co-cultured with or without MSCs and induced them to Treg cells. When CD4^+^ T cells were cultured alone with no MSCs, CD25^+^FoxP3^+^ population was found to be 11.7% in PBMC1 and 11.4% in PBMC2. When they were co-cultured with MSCs, CD4^+^ T cells expressing CD25 and FoxP3 were 23.9% in PBMC1 and 27.1% in PBMC2. Before *in vitro* Treg induction, the percentage of natural Treg cells between PBMC1 and PBMC2 was roughly doubled (1.62% in PBMC1 vs. 3.48% in PBMC2). After Treg induction, no difference in the percentage of CD25^+^FoxP3^+^ Treg cells was found between PBMC1 and PBMC2. Simply, if ICOSL-ICOS signaling promoted Treg proliferation, the percentage of Treg cells in PBMC2 should have been twice as great as that in PBMC1. Based on our findings, we speculate that ICOSL expressed on MSCs likely promotes Treg conversion rather than Treg proliferation through ICOSL-ICOS signaling pathway.

We further revealed that IL-1β, a pro-inflammatory cytokine belonging to the IL-1 family, could induce ICOSL expression in MSCs. Although other inflammatory stimulators such as TNF-α, IFN-γ, and LPS can upregulate ICOSL mRNA in MSCs^21^,^31^, IL-1β was the most potent molecule to induce ICOSL expression in MSCs (Fig. 7a). Furthermore, the induced ICOSL was more stably maintained by IL-1β than by TNF-α, IFN-γ, or LPS in MSCs at both the mRNA and protein levels (unpublished data). Notably, IL-1β pretreated MSCs increased Tregs through ICOSL upregulation, indicating that IL-1β can be used to prime MSCs for potential therapeutic uses. Some clinical studies have reported the beneficial and therapeutic effects of MSCs but others have shown ephemeral benefits or none^47^,^48^,^49^. These conflicting results from clinical studies are driving the search for solutions to improve the efficacy of stem cell therapies^50^. One strategy to attain this goal would be to enlarge the therapeutic window of MSC therapy by activating (priming or licensing) the MSCs before administration. It is difficult to develop a reliable and effective priming method for specific pathological conditions. Our findings demonstrate that IL-1β is a potent priming factor and that IL-1β-primed MSCs show enhanced induction of Tregs to improve the therapeutic efficacy of MSCs by promoting functional Tregs.

Interestingly, MSC preparations show functional variations. Accumulating evidence shows that MSCs obtained by conventional methods are heterogeneous and that there exist various MSC clones with different properties^24^,^25^,^26^. Upon comparing the Treg inducing activity of MSCs using single colony-derived clones, we found that a clone with high basal expression of ICOSL is more effective at Treg induction (Fig. 7). Moreover, BM-derived MSCs were more potent than ASCs in Treg inducing function because BM-derived MSCs expressed more ICOSL compared to ASCs (Supplementary Fig. S5e,f). Collectively, we can extrapolate that the basal ICOSL expression level could be considered one of the potency markers or selection criteria for MSC products with better therapeutic potency.

In this study, we did not include an *in vivo* investigation of ICOSL-expressing human MSCs in animal models. Currently, it is not clear whether ICOSL-expressing human MSCs might xenogeneically promote induction of host Tregs through interactions between human ICOSL and the host ICOS expressed on CD4^+^ T cells. Moreover, we should screen and establish the kind of animal disease models that are suitable for this *in vivo* proof-of-concept study. Although only *in vitro* evidence was described, our findings identify and suggest ICOSL as one of the important factors responsible for the contact-dependent mechanism in MSC-mediated Treg induction. Further studies are required to define and understand the biological significance of ICOSL-mediated Treg induction by MSCs and to achieve optimal improvements in MSC therapy.

## Material and Methods

### Isolation and characterization of MSCs

Human BM aspiration for MSC isolation was approved by the Inha University Hospital Institutional Review Board (IRB number #10–51) and written informed consents were obtained from healthy donors. MSCs were isolated from BM as described previously^51^. For the isolation of clonal MSCs, we used a subfractionation culturing method as described earlier^52^,^53^. The isolated human MSCs were characterized for several cell surface antigens by flow cytometry. The antibodies used for this analysis included anti-CD29 (Serotec, Kidlington, UK), anti-CD44 (Serotec), anti-CD105 (Serotec), anti-CD34 (BD Biosciences, San Diego, CA, USA), anti-CD45 (BD Biosciences), anti-CD90(BD Biosciences), anti-CD73(BD Biosciences), anti-HLA class I (BD Biosciences), anti-HLA-DR (BD Biosciences), anti-CD80 (eBiosciences, San Diego, Cam USA), anti-CD86 (Southern Biotech, Birmingham, AL, USA), and anti-Oct4 (Cell Signaling Technology, Danvers, MA, USA). The cells were analyzed in a flow cytometer (FACSCalibur or FACSVerse; BD Biosciences). Isotype matched control antibodies were used as controls. The MSCs used in this study showed typical MSC marker expression. For ASC isolation, liposuction aspirates were obtained from a healthy female donor after obtaining informed consent (approved by Boondang CHA Hospital, IRB BD2011-152D). All experiments were performed in accordance with the relevant guidelines and regulations. No live animals were used in this study.

### *In vitro* induction of Tregs, co-cultures, and indirect transwell culture

Peripheral blood mononuclear cells (PBMCs) were collected from healthy donors under an Inha University Hospital IRB-approved protocol (IRB #11–104) after obtaining their informed consent. CD4^+^ T cells were purified from PBMCs using a CD4^+^ T cell Isolation Kit (Miltenyi Biotec, Bisley, UK) according to the manufacturer’s instructions. For *in vitro* Treg induction, purified CD4^+^ T cells were cultured in a complete medium containing RPMI1640 (Gibco BRL, Grand Island, NY, USA) supplemented with 10% heat-inactivated fetal bovine serum (FBS; Gibco BRL), 2 mM L-glutamine (Gibco BRL), and 100 U/mL penicillin (Gibco BRL). The wells of a 24-well plate were coated overnight with 1 μg/mL anti-CD3 monoclonal antibody (eBiosciences) at 4 °C. Purified CD4^+^ T cells were seeded at 1 × 10^6^ cells/well and were stimulated with 3 μg/mL anti-CD28 monoclonal antibody (eBiosciences), 1 ng/mL IL-2 (eBiosciences), 5 ng/mL TGF-β (R&D Systems, Minneapolis, MN, USA), and 0.1 μM/mL all-trans-retinoic acid (Sigma-Aldrich, St. Louis, MO, USA) for 2 days. In direct co-cultures, MSCs (1 × 10^5^ cells/well) were co-cultured with CD4^+^ T cells at a ratio of 1:10 (MSCs:CD4^+^). After 2 days, the MSCs were washed thrice with cold phosphate-buffered saline (PBS) supplemented with 0.05 mM ethylenediaminetetraacetic acid (EDTA; Gibco BRL) in order to detach the lymphocytes from MSCs. MSCs were then trypsinized and washed twice with cold PBS-EDTA and stained with a phycoerythrin (PE)-conjugated ICOSL antibody (BioLegend, San Diego, CA, USA) to analyze the ICOSL expression. In transwell cultures, MSCs were seeded in the upper chamber whereas CD4^+^ T cells were seeded in the lower chamber.

### Flow cytometric analysis of Tregs

Flow cytometric analysis was conducted to analyze the phenotypes of the induced Tregs. Cell surface staining was performed with fluorescein isothiocyanate (FITC)- or allophycocyanin (APC)-conjugated CD25 (eBiosciences), APC- or PE-conjugated ICOS (eBiosciences), or FITC-conjugated CD4 (eBiosciences) or PE-Cy7-conjugated CD127 (eBiosciences) for 20 min at 4 °C in the dark. After washing, intracellular staining was performed to detect FoxP3 or IL-10 expression. According to the manufacturer’s instruction, The cells were fixed and permeabilized using Transcription Factor Staining Buffer Set kit (eBiosciences), and then stained with FITC- or PE-conjugated Foxp3 (eBiosciences) or PerCP-Cy5.5- or PE-conjugated IL-10 (eBiosciences). Prior to IL-10 analysis, the cells were stimulated with 40 ng/mL phorbol-12-myristate-13-acetate (Sigma-Aldrich) and 1 μg/mL ionomycin (Sigma-Aldrich) for 5 h. Monensin (Sigma-Aldrich) was added at a concentration of 4 μM for the last 1 h of stimulation.

### Gene expression analysis

Total RNA was extracted from MSCs using the EasyBlue RNA isolation reagent (Intron Biotechnology, Sungnam, Korea). cDNAs were synthesized from 2 μg total RNA using the AccuPower cDNA synthesis kit (Bioneer, Daejeon, Korea). Semi-quantitative reverse transcription-polymerase chain reaction (RT-PCR) was performed using the AccuPower PCR premix (Bioneer) in a thermal cycler (Bio-Rad C1000; Bio-Rad, Hercules, CA, USA). The amplified PCR products were electrophoresed on 1% agarose gels containing SybrSafe (Invitrogen, Carlsbad, CA, USA) and analyzed using a fluorescence image analyzer (LAS4000 mini; Fujifilm, Tokyo, Japan). PCR was performed with the following primers: *IL-10* (forward 5′-ATCCAAGACAACACTACTAA-3′ and reverse 5′-TAA ATATCCTCAAAGTTCC-3′; product 587 bp) and *glyceraldehyde 3-phosphate dehydrogenase (GAPDH*; forward 5′-CCACTGGCGTCTTCACCAC-3′ and reverse 5′-CCTGCTTCACCACCTTCTTG-3′; 476 bp). To confirm the expression of *ICOSL* mRNA, quantitative RT-PCR (qPCR) was performed using the *ICOSL* TaqMan assay (Assay ID: Hs00323621_m1; Applied Biosystems, Waltham, MA, USA) and the TaqMan Universal PCR Master Mix (Applied Biosystems). The mRNA expression level was normalized to *18*S rRNA gene expression (Hs03928985_g1) as an internal control. The qPCR was run on the StepOnePlus real-time PCR System (Applied Biosystems).

### Immunofluorescence staining

After thorough washing with PBS, control or co-cultured MSCs were fixed with 4% paraformaldehyde and permeabilized using 0.5% TritonX-100 (Sigma-Aldrich) dissolved in PBS. The cells were labeled overnight with anti-ICOSL antibody (Santa Cruz Biotechnology) at 4 °C, and then incubated for 1 h with an AlexaFluor488-conjugated secondary antibody (Molecular Probes, Carlsbad, CA, USA). The samples were subsequently stained with 4′,6-diamidino-2-phenylindole (DAPI; Molecular Probes) for 1 min. After mounting, the samples were analyzed by confocal microscopy (Fluoview FV1000, Olympus, Tokyo, Japan).

### Cytokine quantification

Cytokine secretion was measured by enzyme-linked immunosorbent assay (ELISA). According to the manufacturer’s instructions, the amounts of IL-10 and IL-1β were quantified using BD OptEIA (BD Biosciences) and Legend Max (BioLegend) kits, respectively. The conditioned medium from each co-culture was collected followed by measurement of the secreted cytokine. Absorbance was measured on a microplate reader (iMarkmicroplate reader; Bio-RAD).

### ICOSL knockdown and neutralization

For lentiviral shRNA-mediated gene knockdown, the lentivirus was purchased from Santa Cruz Biotechnology (Santa Cruz, CA, USA). MSCs (1 × 10^5^) were seeded onto a 24-well plate. On the next day, adherent MSCs were infected with a control shRNA-bearing lentivirus (shCon) or an ICOSL-shRNA-expressing lentivirus (shICOSL) in the presence of 5 μg/mL polybrene (Sigma-Aldrich) for 24 h. ICOSL knockdown was confirmed by qPCR and flow cytometric analysis. These transduced MSCs were then collected and co-cultured with CD4^+^ T cells under Treg inducing conditions for additional 48 h. For ICOSL neutralization, a neutralizing anti-ICOSL antibody (eBiosciences) was added to the co-cultures at a concentration of 10 μg/mL.

### Overexpression of ICOSL in MSCs

To overexpress ICOSL, 2.5 × 10^6^ 293FT cells (Invitrogen) were seeded in a 100-mm culture dish for lentivirus production. The cells were transfected with ICOSL-expressing pCMV6-AC-GFP vector (Origene, Rockville, MD, USA) in which the full-length human ICOSL gene was cloned along with the packaging constructs (Origene). Two days later, the viral particle-enriched medium was harvested and collected for MSC infection. After MSCs were transduced with the lentiviral particles, ICOSL expression was assessed by qPCR and flow cytometric analyses.

### CFSE proliferation assay

After the co-culture of purified CD4^+^ cells and MSCs for 48 h, the CD4^+^ T cells were harvested followed by further purification using anti-CD25 microbeads to obtain CD4^+^CD25^+^ cells according to the manufacturer’s instructions (Miltenyi Biotech). The CD4^+^CD25^+^ T cells were evaluated for their ability to suppress PBMC proliferation. Briefly, PBMCs (1 × 10^7^ cells in 1 mL of pre-warmed PBS) were labeled with CFSE (Invitrogen) at a final concentration of 10 μM. After incubation at 37 °C for 10 min, the cells were washed with fresh medium containing 10% FBS. The cells labeled with CFSE were stimulated with 3 μg/ mL anti-CD28 antibody (eBiosciences) and co-cultured with isolated Treg cells at a ratio of 1:5 for 3 days. After the co-culture, flow cytometric analysis of cell division by CFSE dilution was conducted to assess PBMC proliferation.

### IL-1β priming

MSCs (or cMSCs) were treated with 10 ng/mL human recombinant IL-1β (R&D Systems) for 24 h. These primed MSCs were harvested and then co-cultured with CD4^+^ T cells for 2 days under Treg inducing conditions. In ICOSL neutralization experiments, a neutralizing anti-ICOSL antibody (10 μg/mL) was added to the co-culture of primed MSCs with CD4^+^ T cells.

### PI3K-Akt inhibition and western blot analysis

For PI3K inhibition, CD4^+^ T cells were pretreated with 5 μM LY294002 (Cell Signaling Technology) for 30 min before Treg induction. For Akt inhibition, pretreatment with GSK690693 (Calbiochem, San Diego, CA, USA) was performed for 30 min at a concentration of 1 μM. At the end of each experiment, CD4^+^ T cells were washed twice with PBS and lysed in lysis buffer (50 mM Tris-HCl, 150 mM sodium chloride, 1 mM EDTA, 1 mM sodium orthovanadate, 1 mM sodium fluoride, 1 mM PMSF, 1% Triton-X 100, and protease and phosphatase inhibitor cocktails (Pierce, Rockford, IL, USA)) for 30 min on ice. Cellular debris was removed by centrifugation and the supernatants were collected. Protein concentration was measured using the BCA protein assay reagent kit (Pierce). Equal amounts of protein were separated by 10% SDS-polyacrylamide gel electrophoresis under reducing conditions and electrotransferred to Immobilon P membranes (Millipore, Billerica, MA, USA). Anti-phospho-Akt (Cell Signaling Technology), anti-Akt (Cell Signaling Technology), and anti-Actin (Santa Cruz Biotechnology) were used for immunodetection. After incubation with an appropriate secondary antibody conjugated to horseradish peroxidase, chemiluminescence was detected in an image analyzer (LAS4000 mini; Fujifilm) using West-Zol Plus (Intron Biotechnology). Actin was used as the loading control. To compare ICOSL protein levels between IL-1β-primed MSCs and cMSCs, the anti-ICOSL (R&D Systems) antibody was used for western blot analysis.

## Additional Information

**How to cite this article**: Lee, H.-J. *et al*. ICOSL expression in human bone marrow-derived mesenchymal stem cells promotes induction of regulatory T cells. *Sci. Rep.* **7**, 44486; doi: 10.1038/srep44486 (2017).

**Publisher's note:** Springer Nature remains neutral with regard to jurisdictional claims in published maps and institutional affiliations.

## Supplementary Material

Supplementary Figure and Data
