# Supplementary material for: ICOSL expression in human bone marrow-derived mesenchymal stem cells promotes induction of regulatory T cells
Source: Sci Rep. 2017 Mar 14;7:44486. doi: 10.1038/srep44486 (PMC5349520; doi:10.1038/srep44486)

Supplementary Figures & Data

ICOSL expression in human bone marrow-derived mesenchymal stem cells promotes induction of regulatory T cells

Hyun-Joo Lee[1,3,*], Si-na Kim[1,2,3,*], Myung-Shin Jeon[1], TacGhee Yi[1,4], Sun U. Song[1,2]

[1] Translational Research Center, Inha University School of Medicine, Incheon, Republic of Korea
[2] SCM Lifesciences Co. Ltd., Incheon, Republic of Korea
[3] Drug Development Program, Department of Biomedical Sciences, Inha University School of Medicine, Incheon, Republic of Korea
[4] SunCreate Co. Ltd., Yangju, Republic of Korea
*These authors contributed equally to this work.

Correspondence and requests for materials should be addressed to T.Y. (email: tgwise@naver.com) or S.U.S.(email: sunuksong@inha.ac.kr)

Supplementary Figure S1

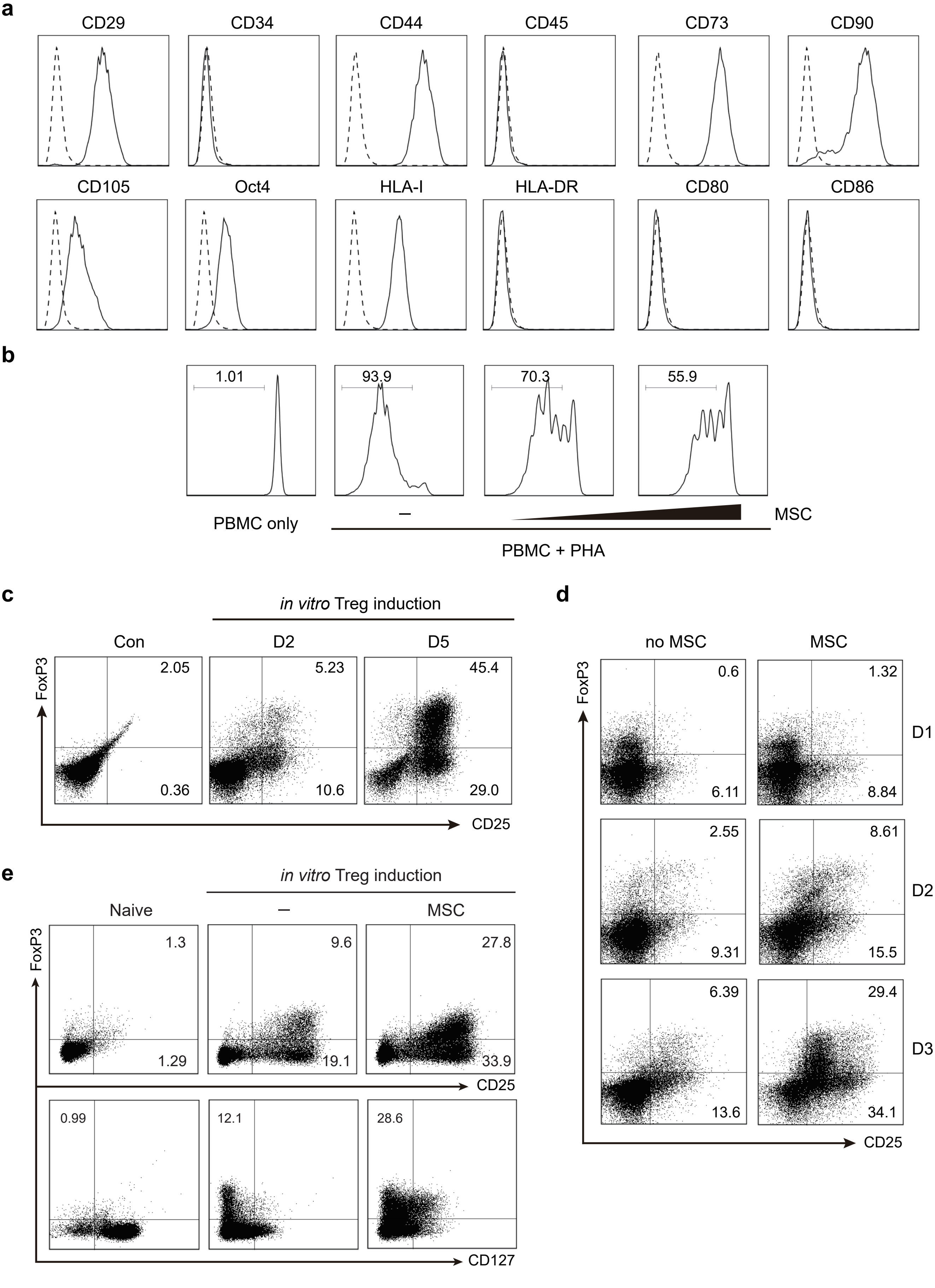

Supplementary Figure S2

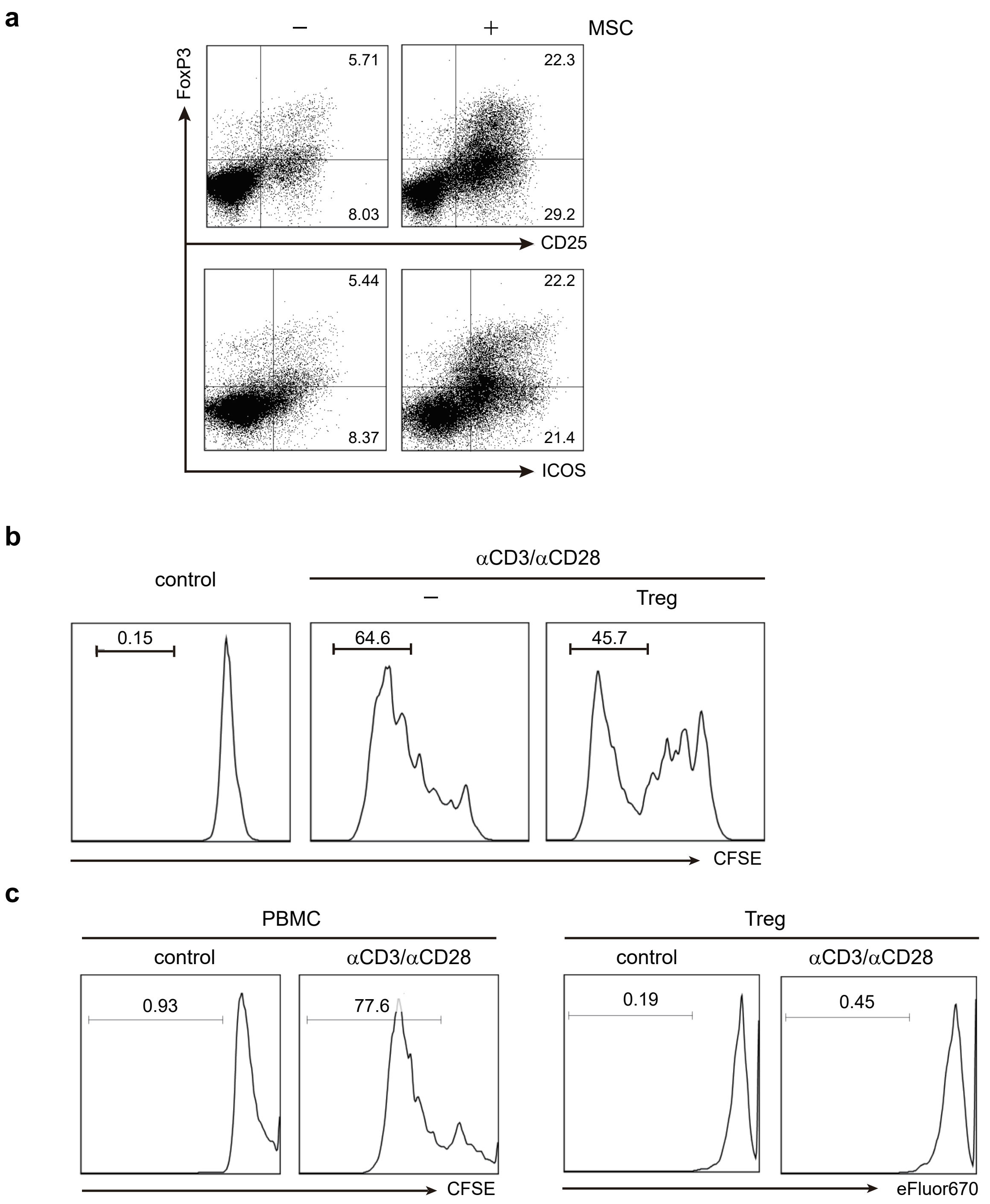

Supplementary Figure S3

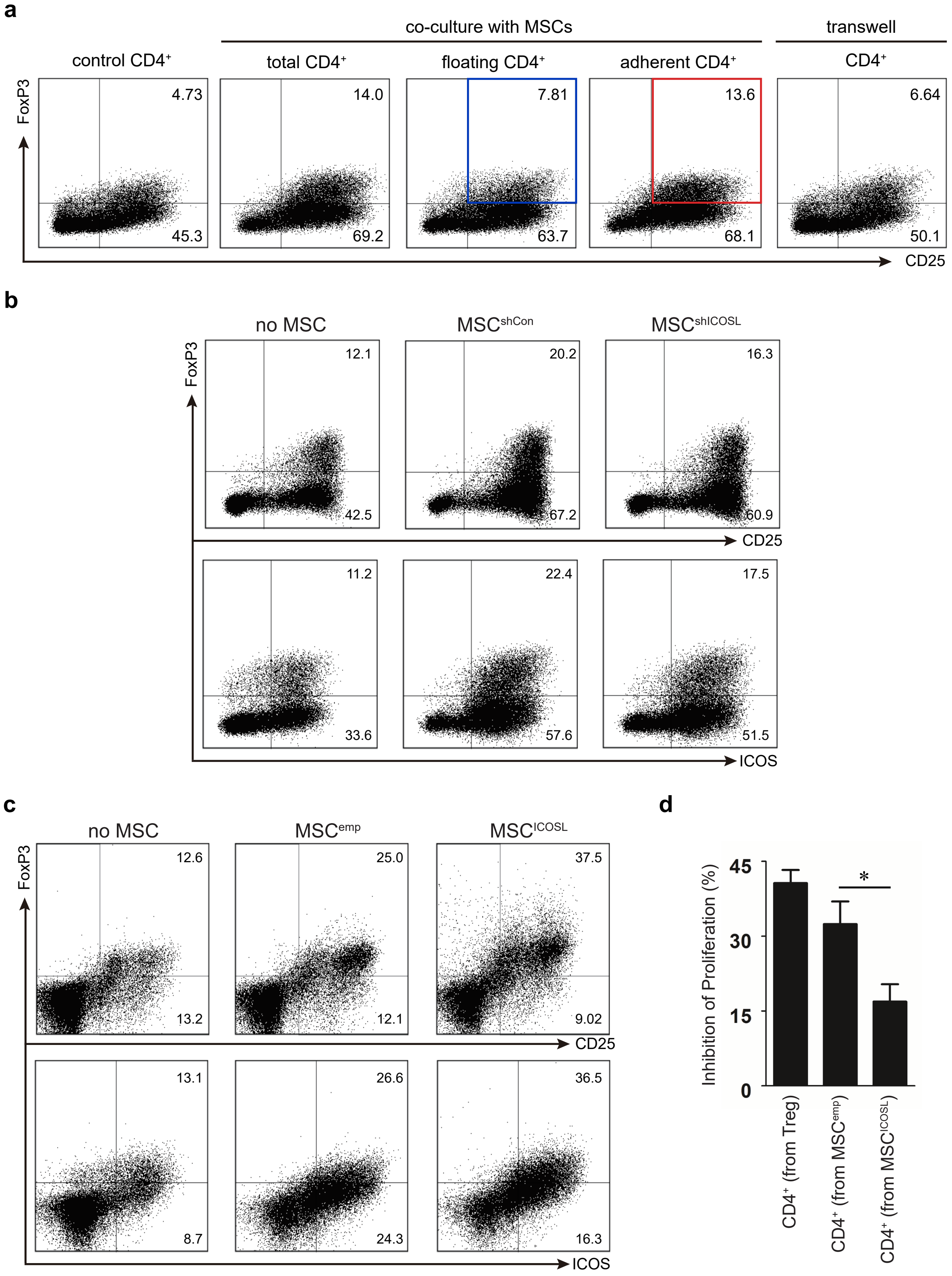

Supplementary Figure S4

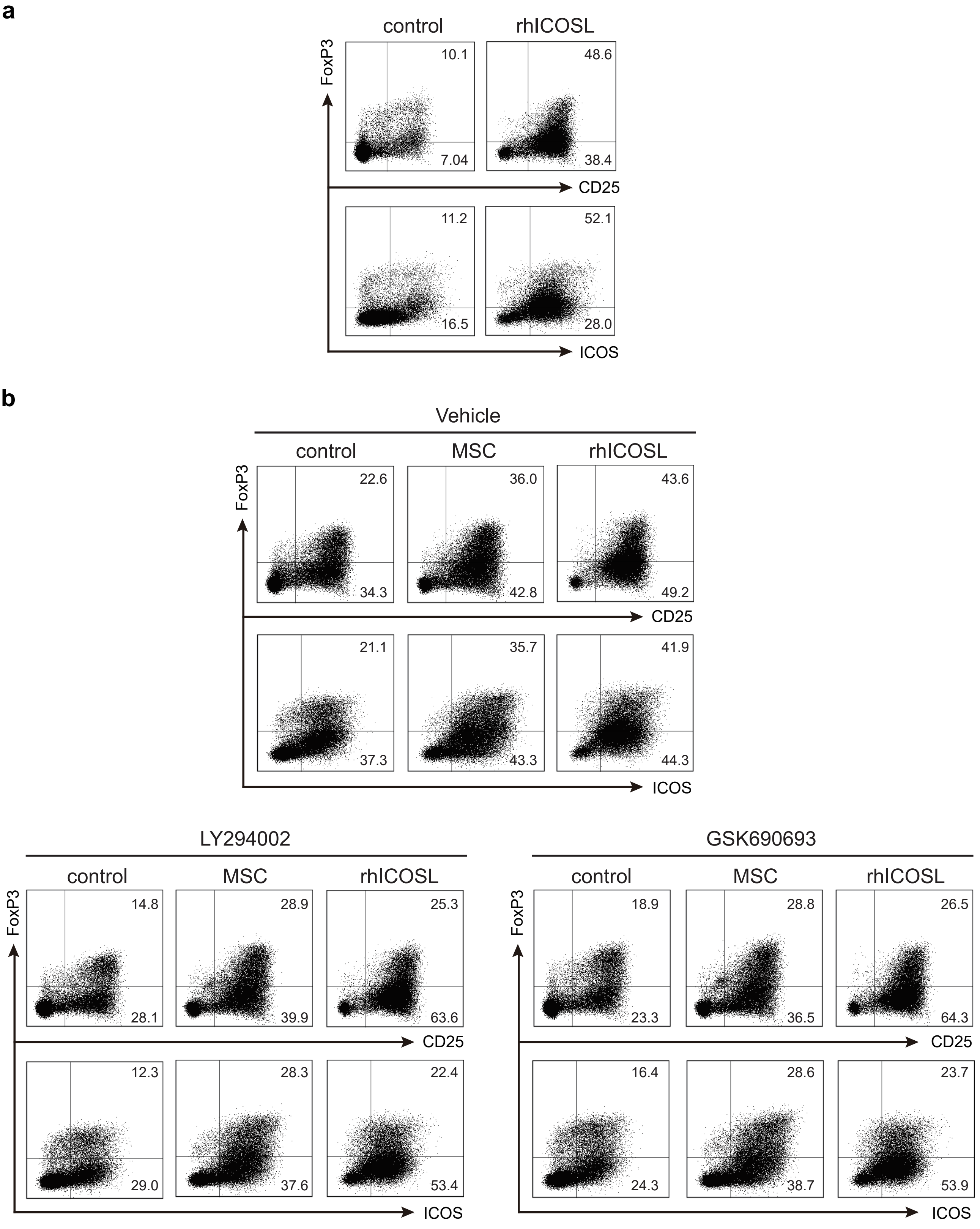

Supplementary Figure S5

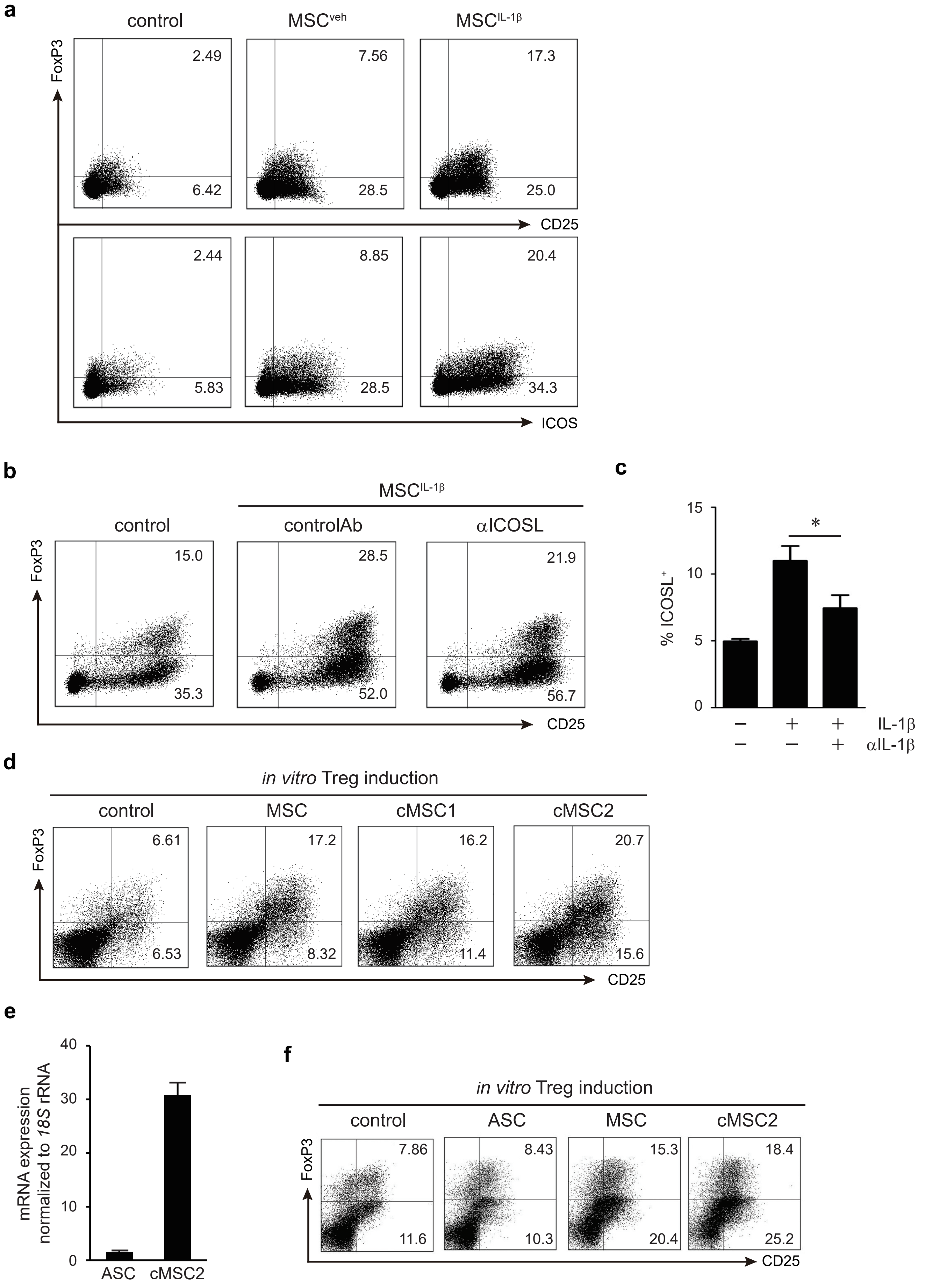

Supplementary Figure S6

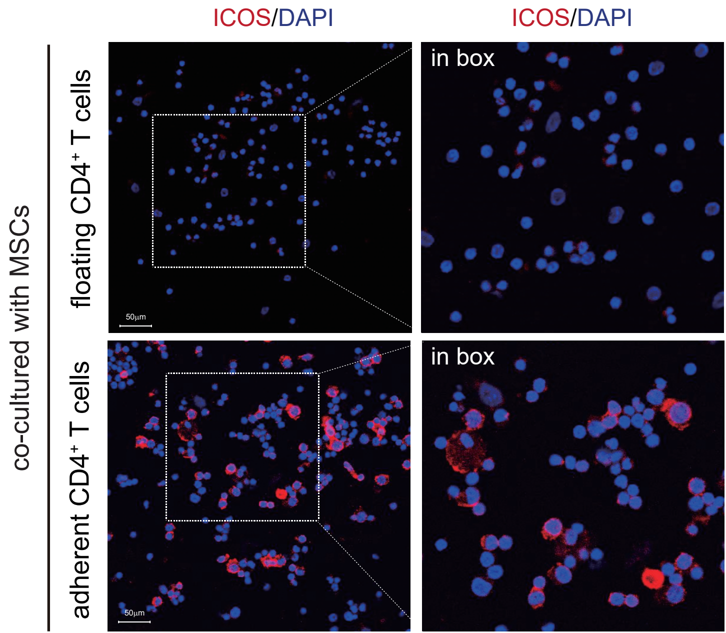

Supplementary Figure S7

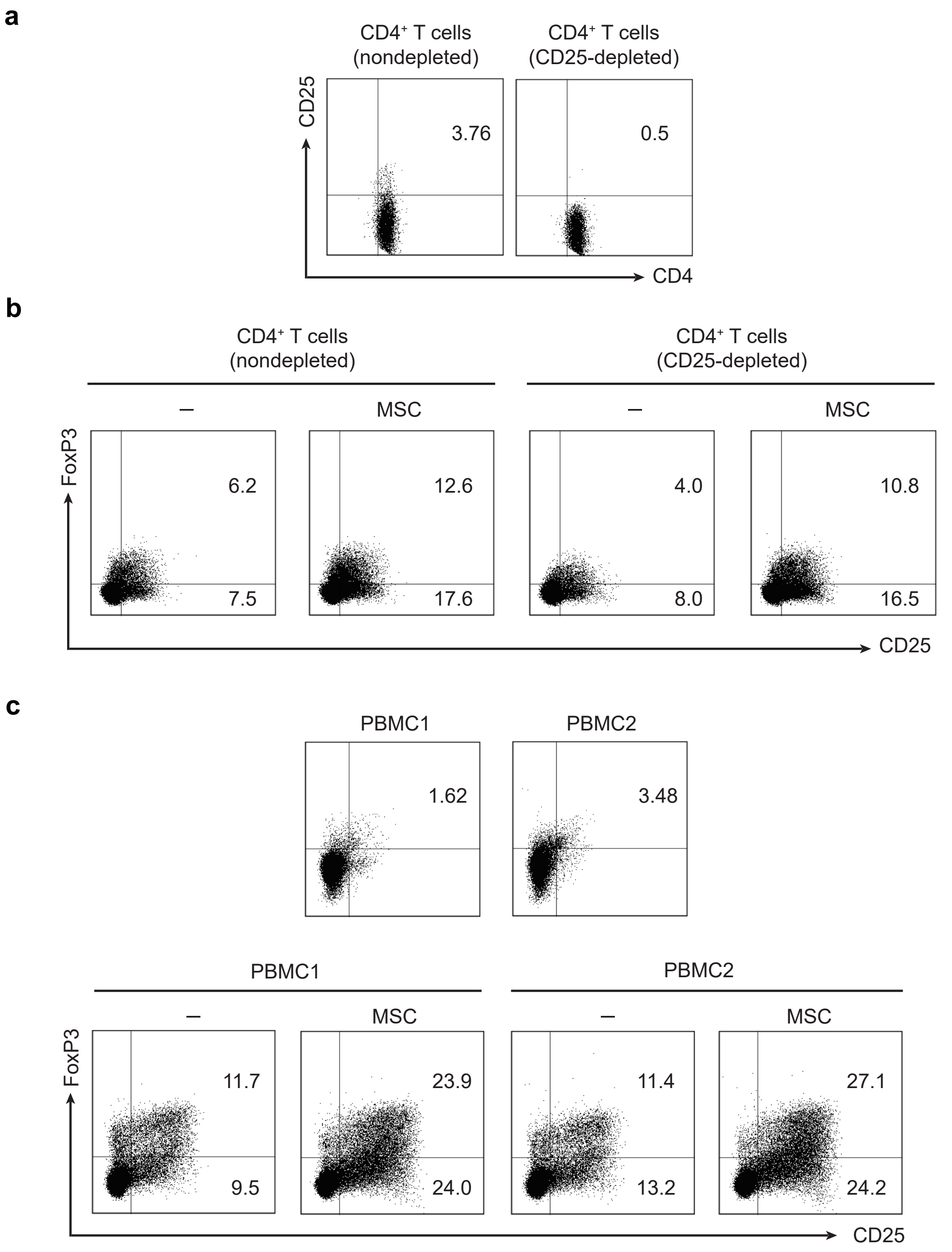

Supplementary Figure Legend

Supplementary Figure S1. (**a**) MSCs used in this study were characterized for marker expression by flow cytometric analysis. Dashed histograms indicate staining with isotype-matched control antibody and solid histograms denote the specific expression of each indicated marker. (**b**) *In vitro* immunosuppressive activity of MSCs was determined by CFSE assay. PBMCs (1×10^6) labeled with CFSE were stimulated with 1 mg/mL PHA in the presence or absence of MSCs (1×10^5 and 2×10^5). After 72 h, PBMCs were harvested and analyzed for their proliferation by flow cytometric analysis. (**c**) For Treg differentiation, $CD4^+$ T cells purified from PBMCs were cultured under *in vitro* Treg inducing condition. Tregs were analyzed by expression of FoxP3 and CD25 up to 5 days. Our *in vitro* Treg induction showed effective induction of Tregs at both day 2 and day 5. (**d**) When MSCs were co-cultured with $CD4^+$ T cells under *in vitro* Treg induction, more $CD25^+$ $FoxP3^+$ Tregs were induced from $CD4^+$ T cells compared. Since there were no difference in Treg induction ratios between day 2 (2.55 vs. 8.61) and day 3 (6.39 vs. 29.4), we chose a 2-day induction protocol throughout this study. **(e)** $CD4^+$ Tregs expressing CD25 and FoxP3 were further analyzed for CD127 expression. CD127 inversely correlated with FoxP3 of human Tregs.

Supplementary Figure S2. (**a**) A representative dot plot of three independent experiments for Fig. 1d. **(b)** MSC-induced Tregs are suppressive. In order to address whether MSC-induced Tregs are completely functional, we performed two *in vitro* tests. MSC-induced Tregs were purified and co-cultured with CFSE-labeled PBMCs at a ratio of 1:10 (Treg:PBMC). After TCR stimulation, we examined whether Tregs inhibits proliferation of activated PBMCs. The result showed that Tregs suppressed proliferation of PBMCs, indicating that MSC-induced Tregs are suppressive. (**c**) MSC-

induced Tregs are anergic. Since anergy is one of the biological properties of Tregs, we then examined whether MSC-induced Tregs are anergic. PBMCs were labeled with CFSE while purified Tregs were lebeled with eFluor670 (eBiosciences) according to manufacturer's instruction. Although TCR stimulation induced proliferation of PBMCs, Tregs did not proliferate in response to TCR stimulation. These results demonstrate that MSC-induced Tregs are both suppressive and anergic.

Supplementary Figure S3. (a) Total $CD4^+$ T cells, only floating T cells, MSC-adherent T cells, or T cells cultured in a transwell plate were subjected to flow cytometric analysis for the $CD25^+FoxP3^+$ Treg phenotype. Non-adherent floating and adherent $CD4^+$ T cells were separately harvested from the same co-cultures. For blue and red boxes, refer to Fig. 2b. Data is a representative of three independent experiments for Fig. 2c. **(b)** When $CD4^+$ T cells were co-cultured with ICOSL knockdown MSCs ($MSCsh^{ICOSL}$), $CD25^+FoxP3^+$ Tregs were significantly decreased compared co-cultures with control MSCs (MSC^{shCon}). Data is a representative of three independent experiments for Fig. 4c. **(c**) Purified $CD4^+$ T cells were co-cultured with MSC^{ICOSL} or MSC^{emp}. Co-culture with MSC^{ICOSL} produced more $CD25^+FoxP3^+$ Treg cells from the $CD4^+$ T cells compared to that in co-culture with MSC^{emp}. MSC^{ICOSL}-induced $CD4^+CD25^+FoxP3^+$ Treg cells were significantly increased. ICOS-expressing Treg cells were also increased upon co-culture with MSC^{ICOSL}. Data is a representative of three independent experiments for Fig. 5c. **(d)** We isolated $CD4^+$ T cells once more from Treg-inducing $CD4^+$ T cells alone, MSC^{emp} co-cultured CD4+ T cells, and MSC^{ICOSL} co-cultured CD4+ T cells and then co-cultured these $CD4^+$ T cells (containing different numbers of Treg population) with activated PBMCs at equal numbers ($CD4^+$ T:PBMC=1:1) for 3 days. Their suppressive activity was determined by inhibition of activated lymphocyte proliferation. In these $CD4^+$ T cell populations, Treg cells were found to be 9.64% in $CD4^+$ T cell alone group, 28.0% in MSC^{emp} co-cultured $CD4^+$ T cell group, and 35.3% in MSC^{ICOSL}

co-cultured $CD4^+$ T cells. As expected, $CD4^+$ T cell population isolated from MSC^{ICOSL} co-culture group showed the most potent inhibitory activity. Data are the average of three independent experiments. Statistical significance was *$P<0.05$.

Supplementary Figure S4. (a) Instead of MSCs, rhICOSL was coated on the plates at a concentration of 5 μg/mL. When $CD4^+$ T cells were seeded and incubated under Treg inducing conditions, increased Tregs were observed with rhICOSL. Data is a representative of three independent experiments for Fig. 6a. **(b)** The effects of PI3K-Akt inhibition on rhICOSL-induced Tregs were examined by incubating rhICOSL-activated CD4+ T cells with LY294002 (LY) or GSK690693 (GSK). After 2 days, CD4+ T cells were analyzed for their expression of CD25, FoxP3, or ICOS by flow cytometric analysis. Data is a representative of three independent experiments for Fig. 6e.

Supplementary Figure S5. (a) MSCs were primed with 10 ng/mL IL-1β ($MSC^{IL\text{-}1\beta}$) or vehicle (MSC^{veh}) for 24 h. The primed MSCs were subjected to co-culture with $CD4^+$ T cells under *in vitro* Treg induction conditions. Flow cytometric analysis was performed to examine the induced Treg phenotype from $CD4^+$ T cells. Data is a representative of three independent experiments for Fig. 7c. **(b)** During Treg differentiation, $CD4^+$ T cells were co-cultured with $MSC^{IL\text{-}1\beta}$ in the presence of 5 μg/mL neutralizing anti-ICOSL antibody. As revealed by flow cytometry, ICOSL neutralization decreased the $MSC^{IL\text{-}1\beta}$-induced Tregs. Data is a representative of three independent experiments for Fig. 7d. **(c)** To confirm the effect of IL-1β on ICOSL upregulation, MSCs were primed with 10 ng/mL IL-1β in the presence or absence of function block anti-IL-1β antibody (10 μg/mL). ICOSL expression in MSCs was measured by flow cytometric analysis. Function block by anti-IL-1β antibody significantly decreased ICOSL-expressing MSCs, suggesting that IL-1β upregulates ICOSL. Data are the the mean ± standard deviation from three independent experiments.

P=0.003. **(d) Treg induction by each MSC population was compared after co-culture with $CD4^+$ T cells under Treg-inducing conditions. Data is a representative of three independent experiments for Fig. 8b. **(e)** ASCs were isolated from liposuction aspirates as desciribed.[1] Relative mRNA expression between BM-derived cMSC2 and ASC was examined by qPCR. **(f)** Treg induction activity of MSC, cMSC2, and ASC was compared after co-culture with CD4+ T cells under in vitro Treg inducing condition. The percentage $CD25^+FoxP3^+$ Tregs induced from $CD4^+$ T cells was analyzed by flow cytometric analysis. Dot blots are a representative of three independent experiments.

Supplementary Figure S6. We performed immunofluorescence staining for ICOS in $CD4^+$ T cells. First of all, $CD4^+$ T cells (1×10^6 cells) and MSCs (1×10^5 cells) were co-cultured under Treg inducing condition for 2 days. Nonadherent floating T cells were harvested by simple pipetting. Next, the remaining cells (MSCs and adherent T cells) were washed three times to remove nonadherent cells. Adherent T cells were detached by thorough and careful pipetting so as not to detach plastic-adherent MSCs. We did not choose trypsinization to detach adherent T cells because it could detach all cells including MSCs. The collected cells (floating and adherent $CD4^+$ T cells) were subject to cytospin (1500rpm for 5 min; Shandon Cytospin 4 Cytocentrifuge, Thermo Shandon). The cells were fixed with 4% paraformaldehyde, blocked with 5% normal goat serum, and incubated with anti-ICOS antibody conjugated with PE (1:100 diluted; BD Pharmingen) overnight at 4^oC. After mounting with aqueous mounting medium with DAPI (Vectashield, Vetctor Laboratories), immunofluorescence was analyzed by confocal microscopy (Fluoview FV1000, Olymphus). In floating $CD4^+$ T cell fraction, ICOS staining was dimly fluorescent around the cell periphery of a few cells. However, a large number of cells were brightly positive for ICOS in adherent fraction, demonstrating that ICOS is upregulated in $CD4^+$ T cells which are in contact with MSCs (*refer to* Fig. 1c for ICOSL-expressing MSCs).

Supplementary Figure S7. (a-b) We performed additional experiments with Treg depleted CD4$^+$ T cells according to Reviewer-#3's suggestion. To obtain the Treg-depleted conventional CD4$^+$ T cells, enriched CD4$^+$ T cells from PBMC3 were depleted of CD25 cells using CD4$^+$CD25$^+$ Regulatory T cell Isolation Kit, Human (Millenyi Biotech). For control, CD4$^+$ T cells were purified from PBMC without CD25 depletion. Depleted or nondepleted CD4$^+$ T cells were induced to Tregs for 2 days. CD25$^+$FoxP3$^+$ cells from CD25-depleted CD4$^+$ cells alone were 4.0% while CD25$^+$FoxP3$^+$ cells from nondepleted CD4$^+$ cells alone were 6.2%. When co-cultured with MSCs, CD25-depleted CD4$^+$ cells generated 10.8% of CD25$^+$FoxP3$^+$ cells and nondepleted CD4$^+$ cells produced 12.6% of CD25$^+$FoxP3$^+$ cells. Our data showed that Treg-depleted conventional CD4$^+$ T cells generated as many Treg cells as nondepleted CD4$^+$ T cells did. **(c)** We simply purified CD4$^+$ T cells (1×10^6 cells/each) from 2 different PBMCs (named PBMC1 and PBMC2) without Treg depletion. At that time, CD4$^+$ T cells expressing CD25 and FoxP3 were 1.62% in PBMC1 and 3.48% in PBMC2 by flow cytometric analysis. Each CD4$^+$ T cell population was co-cultured with or without MSCs (1×10^6 cells) and induced them to Treg cells for 2 days. When CD4$^+$ T cells were cultured alone with no MSCs, CD25$^+$FoxP3$^+$ population was found to be 11.7% in PBMC1 and 11.4% in PBMC2. When they were co-cultured with MSCs, CD4$^+$ T cells expressing CD25 and FoxP3 were 23.9% in PBMC1 and 27.1% in PBMC2. Before *in vitro* Treg induction, the percentage of natural Treg cells between PBMC1 and PBMC2 was roughly doubled (1.62% in PBMC1 vs. 3.48% in PBMC2). After Treg induction, no difference in the percentage of CD25$^+$FoxP3$^+$ Treg cells was found between PBMC1 and PBMC2.

Supplementary Data

a. Original gel images for Fig. 3d

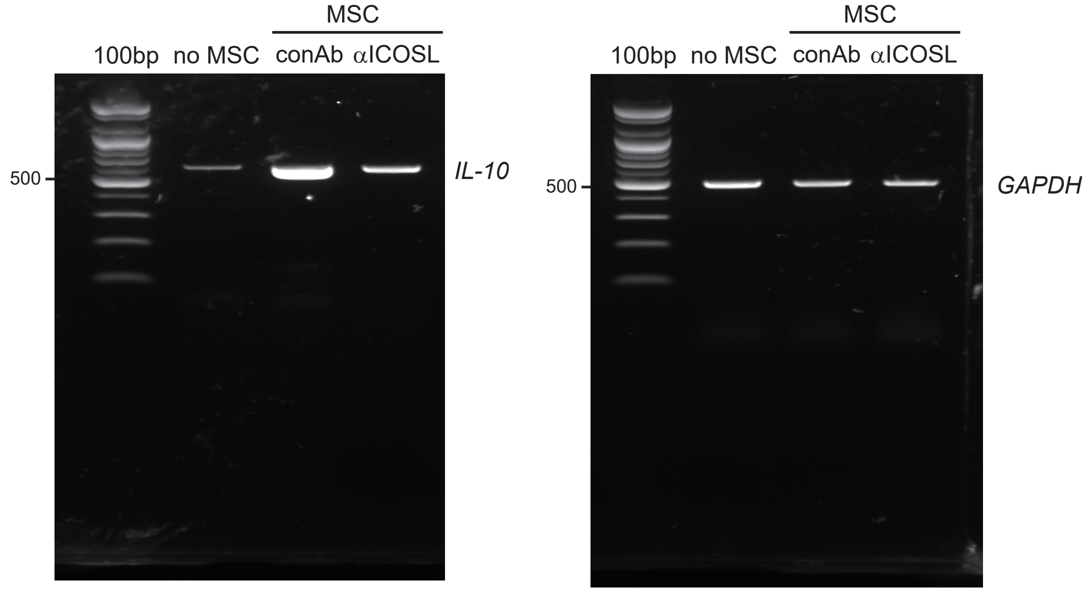

b. Original gel images for Fig. 5d

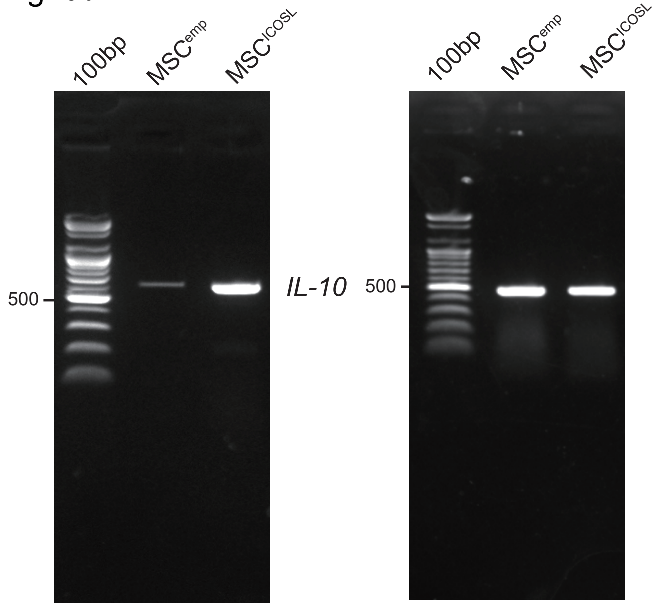

c. Original blot images for Fig. 6b

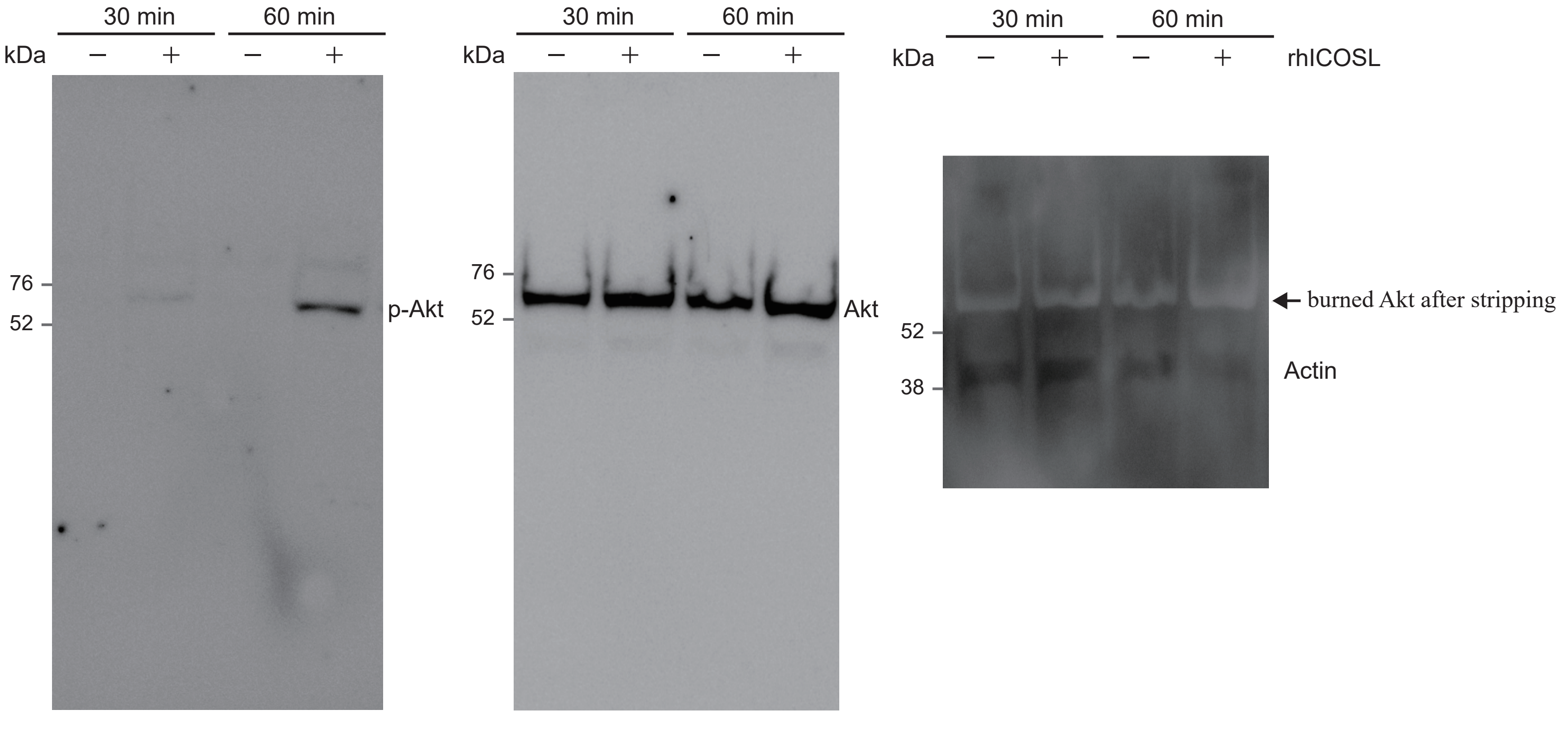

d. Original blot images for Fig. 6c

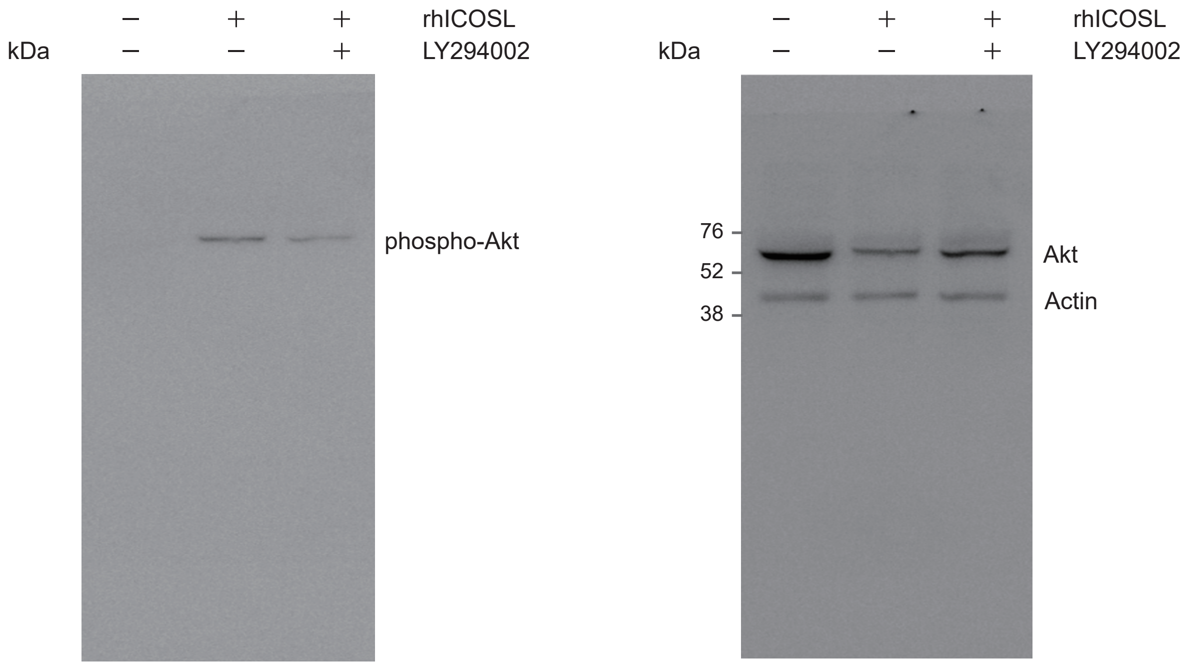

e. Original blot images for Fig. 6d

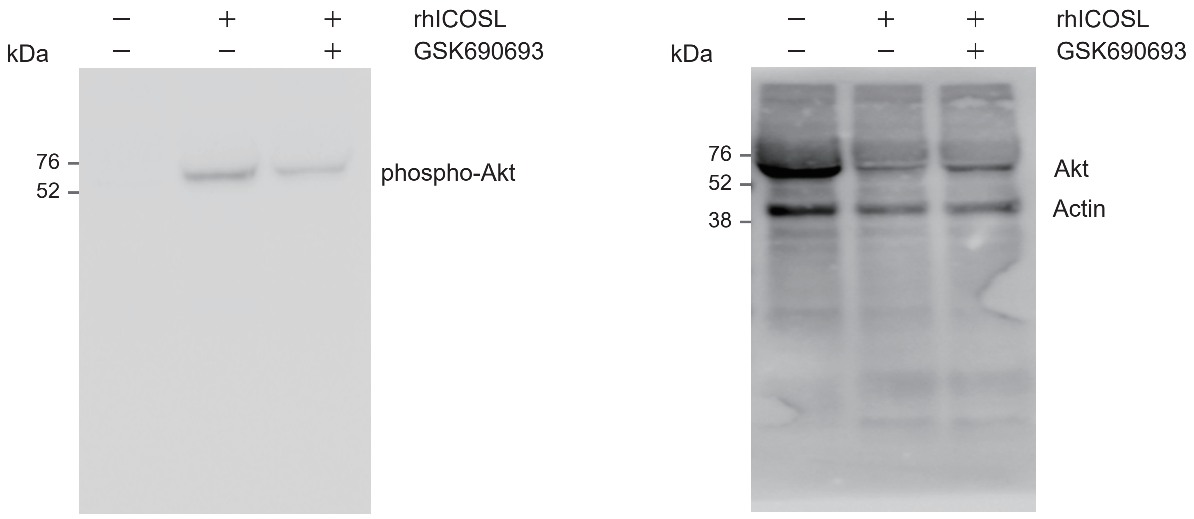

f. Original blot images for Fig. 8d

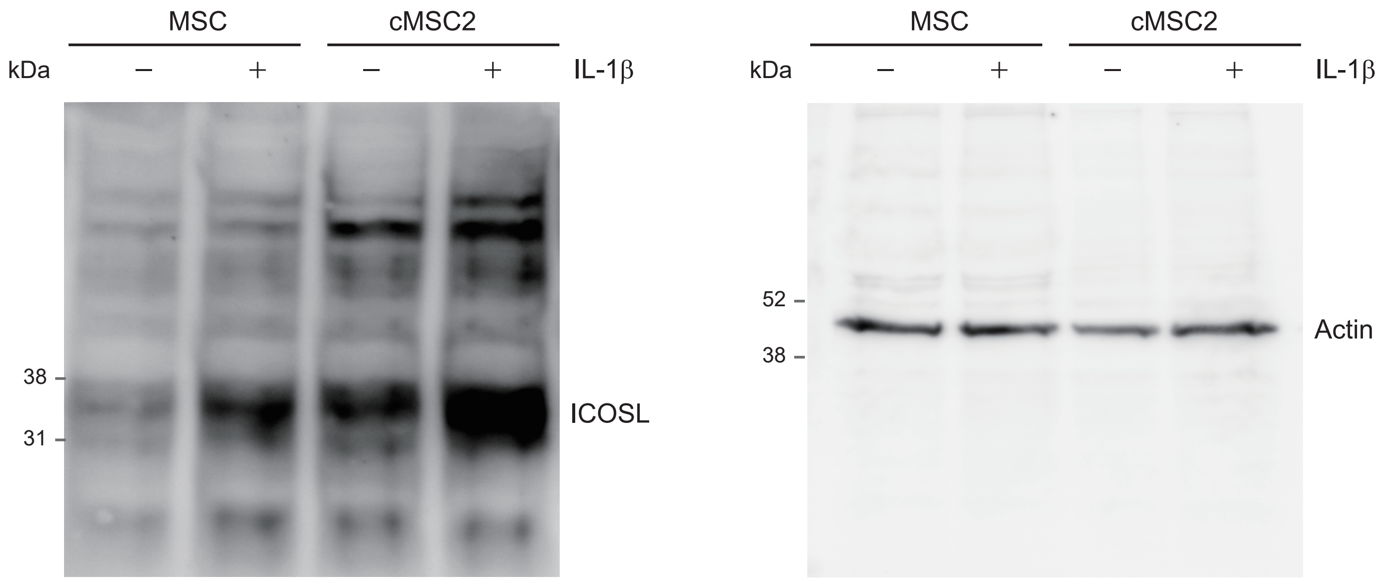

Supplement: Supplementary Figure and Data [file srep44486-s1.pdf]
